# Predictive Ability of Previous Pain and Disease Conditions on the Presentation of Post‐COVID Pain in a Danish Cohort of Adult COVID‐19 Survivors

**DOI:** 10.1002/ejp.70021

**Published:** 2025-04-05

**Authors:** Brian Duborg Ebbesen, Jakob Nebeling Hedegaard, Simon Grøntved, Rocco Giordano, César Fernández‐de‐las‐Peñas, Lars Arendt‐Nielsen

**Affiliations:** ^1^ Department of Health Science and Technology, School of Medicine, Center for Neuroplasticity and Pain Aalborg University Aalborg Denmark; ^2^ Department of Gastroenterology & Hepatology, Mech‐Sense Clinical Institute, Aalborg University Hospital Aalborg Denmark; ^3^ Department of Clinical Medicine, Danish Center for Health Services Research Aalborg University Aalborg Denmark; ^4^ Region North Psychiatry Aalborg University Hospital Aalborg Denmark; ^5^ Department of Oral and Maxillofacial Surgery Aalborg University Hospital Aalborg Denmark; ^6^ Department of Physical Therapy, Occupational Therapy, Physical Medicine and Rehabilitation Universidad Rey Juan Carlos (URJC) Madrid Spain; ^7^ Steno Diabetes Center North Denmark Clinical Institute, Aalborg University Hospital Aalborg Denmark

## Abstract

**Background:**

Even though many post‐COVID pain risk factors have been identified, little is known about the predictive profiles of these risk factors for the development of post‐COVID pain.

**Methods:**

Data was collected from two separate questionnaires assessing demographics, pre‐existing medical comorbidities, pain history, and post‐COVID pain experience. Socioeconomic data and COVID‐19 RT‐PCR test results were collected from Danish registries. The study cohort (*n* = 68,028) was stratified into two groups reporting pre‐COVID pain (*n* = 9090) and no pre‐COVID pain (*n* = 55,938). Forward‐selection prediction models were employed to identify predictor profiles for post‐COVID pain in the full study cohort (Model 1) and the stratified groups with (Model 2) and without (Model 3) pre‐COVID pain from 58 potential risk factors.

**Results:**

Model 1 achieved a 5‐fold cross‐validated AUC (cvAUC) of 0.68. Use of pain medication, stress, high income, age, female gender, and weight were the top predictors contributing to 97% of the model performance. Model 2 (cvAUC = 0.69) identified use of pain medication, breathing pain, stress, height, physical activity, and weight as the top predictors contributing to 98.6% of model predictive performance. Model 3 (cvAUC = 0.65) identified stress, female gender, weight, higher education, age, high income, and physical activity as the top predictors contributing to 98.5% of model predictive performance. Height was unique to Model 2, while being female and higher income were unique to Model 3.

**Conclusions:**

The study highlights potential important predictors, and further research is needed to describe these in detail. The results may apply to the understanding of post‐viral pain sequelae after other viral infections.

**Significance Statement:**

The explorative study investigates the predictive ability of a battery of pre‐COVID risk factors potentially associated with the development of post‐COVID pain. This article presents the profiles of predictors of interest in COVID‐19 survivors with and without pre‐COVID pain. The results will contribute to the understanding of patient profiles that might develop post‐COVID pain conditions and provide a first step towards focused clinical predictive research.

## 1 Introduction

Five years into the Severe Acute Respiratory Syndrome Coronavirus 2019 2 (SARS‐CoV‐2) pandemic, the coronavirus disease 2019 (COVID‐19) emerged as a global health crisis, post‐COVID pain, among a plethora of persisting symptoms within long‐COVID, stands out as one prominent feature (Davis et al. 2023a; Donnachie et al. 2022; Fernández‐de‐las‐Peñas, Nijs, et al. 2022; Fernández‐de‐las‐Peñas, Navarro‐Santana, et al. 2022; Rochmawati et al. 2024; Shabnam et al. 2023; Sørensen et al. 2022). Post‐COVID pain studies indicated that up to 20% of COVID‐19 survivors up to 2 years after the initial infection experience one or more pain symptoms (Castaldo et al. 2023; Fernández‐de‐las‐Peñas, Liew, et al. 2022; Fernández‐de‐las‐Peñas, De‐la‐Llave‐Rincón, et al. 2022; Kerzhner et al. 2024).

Recent research has highlighted de novo pain as an important entity of persistent post‐COVID‐19 conditions based on self‐reported data (Castaldo et al. 2023; Kerzhner et al. 2024; O'Mahoney et al. 2023; Zeng et al. 2023) and patient records (Reese et al. 2023). A growing body of large cohort studies has revealed several potential risk factors of post‐COVID pain experience (Ebbesen, Giordano, Valera‐Calero, et al. 2024; Ebbesen, Giordano, Hedegaard, et al. 2024). Female sex (Atchison et al. 2023; Crook et al. 2021; Hastie et al. 2022, 2023; Michelen et al. 2021; Notarte et al. 2022; Sørensen et al. 2022; Thompson et al. 2022), older age (Atchison et al. 2023; Crook et al. 2021; Donnachie et al. 2022; Hastie et al. 2022, 2023; Michelen et al. 2021; Thompson et al. 2022), socioeconomic (educational and income levels) (Jakobsen et al. 2023; Shabnam et al. 2023; Subramanian et al. 2022; Thompson et al. 2022) and psychological factors (mainly depression, anxiety, and stress) (Davis et al. 2023a; Donnachie et al. 2022; Greißel et al. 2024; Hastie et al. 2022; Jacobs et al. 2023), and pre‐COVID conditions (Crook et al. 2021; Hastie et al. 2022; Jacobs et al. 2023; Notarte et al. 2022; Pretorius et al. 2022) are among the most prominent risk factors for post‐COVID pain experience outcomes in previously hospitalised (Ebbesen, Giordano, Valera‐Calero, et al. 2024; Fernández‐de‐las‐Peñas, De‐la‐Llave‐Rincón, et al. 2022; Fernández‐de‐las‐Peñas, Martín‐Guerrero, et al. 2023) and non‐hospitalised cohorts (Bell et al. 2021; Ebbesen, Giordano, Hedegaard, et al. 2024; Sørensen et al. 2022). While the SARS‐CoV‐2 infection's effects are recognised, various endogenous and exogenous factors (Matta et al. 2022; Selvakumar et al. 2023; Zeng et al. 2023) and comorbidities are all contributing risk factors (Chen et al. 2022; Gevers‐Montoro et al. 2023; Hua et al. 2024; Russell et al. 2023).

Pre‐COVID pain conditions might be further exacerbated after the infection (Ebbesen, Giordano, Valera‐Calero, et al. 2024; Ebbesen, Giordano, Hedegaard, et al. 2024) and lead to additional post‐COVID pain symptomatology (Castaldo et al. 2023). Also, the presence of pre‐COVID pain of chronic nature, and in particular pain with nociplastic features such as chronic overlapping pain and musculoskeletal pain conditions and fibromyalgia, is a well‐documented risk factor for experiencing de novo post‐COVID pain (Bergmans et al. 2024; Fernández‐de‐las‐Peñas, De‐la‐Llave‐Rincón, et al. 2022; Fernández‐de‐las‐Peñas et al. 2024; Kerzhner et al. 2024; Subramanian et al. 2022; Thompson et al. 2022). Thus, having a pain condition pre‐infection may lead to an exacerbation of existing pain after the infection (Fernández‐de‐las‐Peñas, Florencio, et al. 2021, Fernández‐de‐las‐Peñas, De‐la‐Llave‐Rincón, et al. 2022, Fernández‐de‐las‐Peñas, Raveendran, et al. 2023). However, the identification of relevant factors for predicting post‐COVID pain remains a challenge due to the highly variable and heterogeneous nature of symptoms (Fernández‐de‐las‐Peñas, Liew, et al. 2022; Fernández‐de‐las‐Peñas, Raveendran, et al. 2023; Hayes et al. 2021; Hua et al. 2024; Jacobs et al. 2023; Kerzhner et al. 2024). Furthermore, risk factors only tell part of the story as they do only establish an increased probability of an event based on a certain condition. A predictive approach, on the other hand, provides a way of identifying people at elevated risk of developing post‐COVID pain from the already known risk factors under investigation (Schooling and Jones 2018) Additionally, meta‐analyses of risk factors report conflicting results (Fernández‐de‐las‐Peñas, Raveendran, et al. 2023; Notarte et al. 2022; Perumal et al. 2023) thus, little is known about the predictive value of the individual risk factors (Hua et al. 2024; Jacobs et al. 2023; Prosepe et al. 2022). Therefore, it would be of importance in a large cohort to investigate if the profile of predictive factors for the presentation of post‐COVID pain differs between those with pre‐COVID pain and those without. By applying a basic explorative predictive modelling approach, the current research could create a better understanding of the most actionable risk factors to enable a future, more casual approach with a proper application value to benefit clinicians helping COVID‐19 survivors. Furthermore, the current study could help mature the field of post‐COVID pain in relation to applying more advanced prediction models focusing on the many possible interaction effects.

The aims of this large nationwide cohort study were (1) to explore the predictive value of an extensive array of potential pre‐COVID risk factors for the presentation of general post‐COVID pain and (2) to investigate potential differences in profiles of predictive risk factors in people who had pre‐COVID pain and those who had no pre‐COVID pain.

## 2 Methods

### 2.1 Study Design

This study was an explorative, questionnaire‐based, prediction‐driven investigation of self‐reported post‐COVID pain features in a Danish cohort of people with a positive reverse transcription‐polymerase chain reaction (RT‐PCR) test between 1 March 2020 and 31 December 2021. The social security number (CPR) for each participant was used to extract data from the national Danish COVID‐19 surveillance system based on permission granted by Statens Serum Institut and the Danish Health Data Agency (approval number FSEID‐00006572). The CPR uniquely identifies all citizens in Denmark (Mainz et al. 2019). The study was primarily based on self‐reported questionnaire data. A standardised questionnaire (Questionnaire 1) was administered to all participants collecting (1) socio‐demographics, (2) pre‐COVID comorbidities, (3) pre‐COVID pain conditions, (4) de novo post‐COVID pain experience, (5) pain intensity on a 4‐point Likert scale from mild to very severe, and (6) pre‐COVID pain medication use. Participants were excluded if CPR, weight, height, or age were not available or considered outliers, or if any data were missing. A detailed description of the questionnaire can be found in previous work (Ebbesen, Giordano, Hedegaard, et al. 2024). Data accrual started and ended on 22 August 2022 and 23 September 2022, respectively. The collected questionnaire data was linked to COVID‐19 surveillance data maintained by Statens Serum Institute and registries containing (1) socioeconomic data about income and educational level, (2) cohabitant status, and (3) information about resident children maintained by Statistics Denmark (Gregersen et al. 2018). A follow‐up questionnaire (Questionnaire 2) was sent out to collect baseline data from participants not reporting post‐COVID pain as these data were not collected for this cohort in Questionnaire 1. The study was approved by the Danish Data Protection Agency (approval number F2022‐004) and complied with the Danish Health Data Act (approval number 2022‐056227). The Danish legislation did not require ethical approval from the Scientific Ethics Committee to conduct the survey. The data have previously been used for other purposes in two publications (Ebbesen, Giordano, Valera‐Calero, et al. 2024; Ebbesen, Giordano, Hedegaard, et al. 2024).

### 2.2 Participants

**FIGURE 1:**
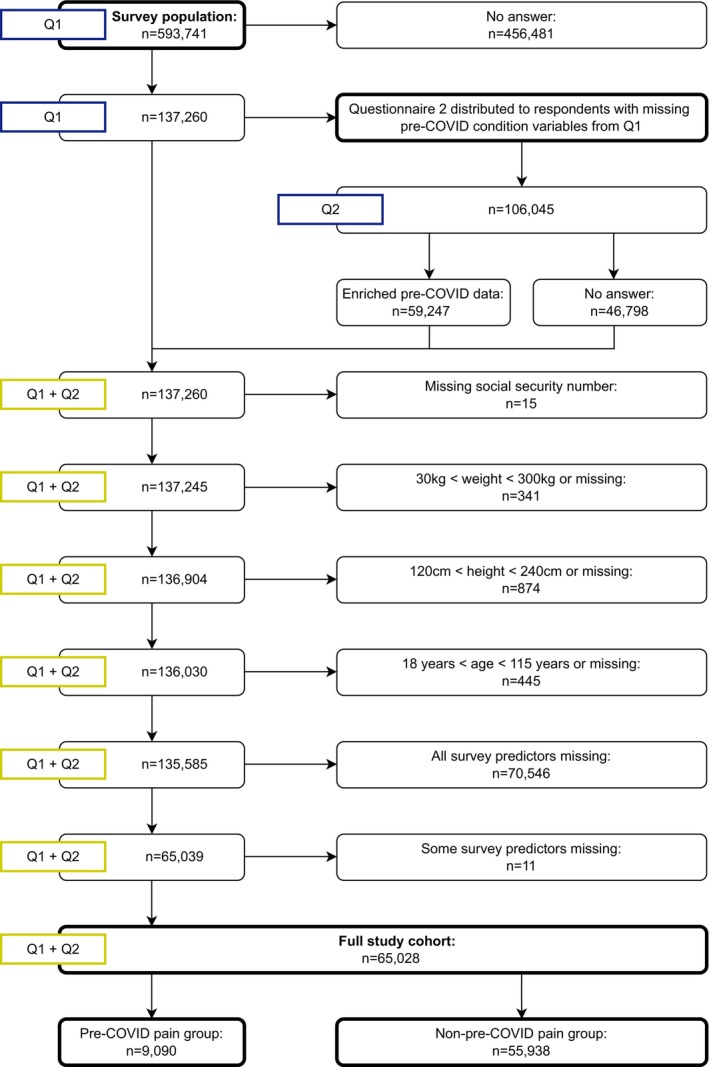
Population description showing the number of participants based on the different exclusion criteria.

The questionnaire was distributed to 593,741 adult Danish residents at the time of the questionnaire. It was distributed if participants had: (1) a valid CPR, (2) an RT‐PCR‐verified history of SARS‐CoV‐2 infection on 31 December 2021, (3) consented to participate in the study via informed consent, and (4) access to the official, Danish, national, secured, mailing system e‐Boks (Digitaliseringsstyrelsen 2022). Informed consent was collected via the first question in the questionnaire. If the participant did not provide informed consent, the questionnaire would end with no further action required after the initial question. Participants were included in the full study cohort if they had completed both Questionnaires 1 and 2. To address the first aim, the full study cohort is defined as all participants included in the current study and is shown in detail in Figure 1.

### 2.3 Variables

The outcome was defined as post‐COVID pain characterised by participants (1) with pre‐COVID pain in general reporting new pain and/or (2) reporting de novo post‐COVID pain experience and was obtained from questionnaire 1. Variables were obtained from both the questionnaires and registries. To investigate the profile of predictive factors according to the second aim, the full study cohort was stratified into two subgroups, (1) a pre‐COVID pain group and a non‐pre‐COVID pain group. The subgroup stratification depended on whether the participants self‐reported general pre‐COVID pain in Questionnaire 1. The variables extracted from the questionnaire data included basic physiological factors (height, weight from where Body Mass Index (BMI) was calculated), pain information (type of previous pain, use of pain medication if any, number of pain types, and if they developed general post‐COVID pain according to the outcome definition), level of daily physical activity, smoking status, and comorbidities. From the registries, we obtained gender (referred to in the variables as ‘female (CPR)’, where CPR is the Danish social security number), date of birth, cohabitation status, number of children living with the participant, income, and highest achieved educational level. Age was defined as age at the beginning of the initial data acquisition (22 August 2022). Income was defined as accumulated income between 2018 and 2020. Educational levels were recorded as International Standard Classification of Education (ISCED) levels, and grouped into “Low”, “Medium” and “High” corresponding to ISCED levels 0–2, 3–4, and 5–8 respectively. Continuous variables have been categorised to allow for greater interpretation of the data by sacrificing some performance effectivity to improve overall model performance and reliability of the results (Barrio et al. 2017). Even though a categorization might overrate the predictive ability of a model when applied to new data, such a manoeuvre provides a better idea of which kind of people in the full study cohort are investigated. For a complete list of variables, their sources, categorizations, and definitions, see Tables S1 and S2. Income and educational level had a low, but nonnegligible amount of missingness in the full study cohort which was handled in the prediction models by including missingness as a separate category.

### 2.4 Statistical Analysis

The full study cohort was characterised in terms of the baseline characteristics using standard summary statistics. For categorical variables, proportions and counts associated with each category were reported, and continuous variables were summarised in terms of medians and Inter Quartile Range (IQR). In the predictive models, age (in years) and BMI (kg/m^2^) were categorised in the intervals: 18–40, 40–60, and 60–80 years, and 0–25, 26–30, 31–35, and 36–40 kg/m^2^, respectively. This categorisation was introduced in the model to help describe the population included in this model. The intervals are referred to in brackets throughout this study (i.e., the variable that encompasses ages 40 to 60 is referred to as age [40,60]). The proportions and counts for these binned predictors were reported.

A total of 58 potential predictors were chosen for the prediction models. Before the modelling, a rudimentary sample size calculation was performed to find a suitable maximum number of predictors. To include 58 potential predictors, the sample size for the prediction models was calculated to be a minimum of 29,042 participants (Riley et al. 2020). To achieve a list of potential predictors of interest, we prioritised the entire range of variables using forward selection. This was done by greedily forward‐selecting the predictors using *p*‐values in logistic regression models (Hastie et al. 2008). At each step of the forward selection, univariate and multivariate coefficients, apparent area under the curves (AUC), and 5‐fold cross‐validated AUCs (cvAUC) were calculated. The cvAUCs were used as stopping criteria, terminating the models at the first step at which the cvAUC decreased. To account for possible multicollinearity effects, the distribution of the rankings of the individual variables was bootstrapped using 200 bootstrap samples of each of the three cohorts. The prediction models included potential predictors focusing on the predictive performance of these. Thus, for the categorised variables age and BMI as well as the continuous variables weight and height, the intention was not to explain if they show protective features or not. Rather, the models served to highlight potential predictors of interest for future research with a more specific clinical focus on the distinct features. Even though the model in principle allowed for the usage of interaction effects between predictors, the justification for this was not found to be solid enough, given the limited knowledge about the predictive factors themselves. Furthermore, due to the large number of possible interactions (1653 for the full study cohort alone) it would not be possible to objectively prioritise these and select only those of clinical relevance due to the relative infancy of the field of post‐COVID pain prediction.

The current study was explorative with the intention of investigating variables that may be of interest to future studies on post‐viral infection sequelae. Thus, the prediction models did not require external validation. To investigate the predictor profiles in the 2 subgroups (pre‐COVID pain group versus the non‐pre‐COVID pain group) for the post‐COVID pain outcome, the same procedure was applied as in the main analysis by (1) forward‐selecting the entire range of variables using *p*‐values in a logistic regression model, (2) calculating univariate and multivariate coefficients, and (3) calculating apparent and 5‐fold cvAUCs. Pre‐COVID pain variables were excluded from the pre‐COVID pain group as this was the stratification factor. This approach resulted in three models: Model 1: the full study cohort model based on data from the full study cohort of 65,028 mixed participants where the outcome is further defined as a presentation of post‐COVID pain, Model 2: the pre‐COVID pain group model based on data from 9090 participants reporting pre‐COVID pain with an exacerbation of existing pain or new post‐COVID pain experience as outcome, and Model 3: the non‐pre‐COVID pain group model based on data from the 55,938 participants not reporting pre‐COVID pain where the post‐COVID pain outcome is further defined as de novo post‐COVID pain experience. Results of the forward‐selection process for each of the Models 1–3 were plotted as a function of the 58 potential predictors and the apparent and cvAUC showing the increased predictive performance after each step. The stopping point was marked with a dotted line for clarity. Finally, the performance of each model was visualised as receiver operating characteristic (ROC) curves, depicting the specificity and sensitivity of each model as the integral under each ROC curve.

## 3 Results

Questionnaire 1 was distributed to 593,741 Danish adults with a previous positive RT‐PCR test. A total of 137,260 responses were returned. Later, due to various restrictions of Questionnaire 1, the baseline characteristics of participants without self‐reported post‐COVID pain were not obtained. Thus, questionnaire 2 was reissued as a follow‐up questionnaire on 28 April 2023, with data accrual ending on 2 June 2023. Questionnaire 2 was distributed to the 106,045 cohort participants from Questionnaire 1 who reported no post‐COVID pain. Individuals with missing or outlying data were excluded (see Figure 1). Thus, the final cohort consisted of 65,028 COVID‐19 survivors (57.6% female, median (CI) 56.4 (46.2–65.6)) with a confirmed SARS‐CoV‐2 infection between 1 March 2020 and 31 December 2021. Registered socioeconomic data of participants was collected from the Danish registries, with 0.3%–1.3% missing depending on the variable, see Table 1.

**TABLE 1:** Demographic and sociodemographic data of participants stratified into the full study cohort, the pre‐COVID pain group, and the non‐pre‐COVID pain group. The pre‐COVID pain group reported one or more pre‐COVID pain conditions, while the non‐pre‐COVID pain group did not have pre‐COVID pain.

	Full study cohort (*n* = 65.028)	Pre‐COVID pain group (*n* = 9090)	Non‐pre‐COVID pain group (*n* = 55,938)
Post‐COVID pain	17.7% (11,535)	33,7% (3063)	15.1% (8472)
Female, % (*N*)	57.6% (37,456)	70.9% (6447)	55.4% (31,009)
Age at first questionnaire, median (IQR)	56.4 (46.2–65.6)	55.9 (46.1–64.4)	56.5 (46.2–65.9)
Age at first questionnaire (categorised), % (*N*)			
18–40	15.4% (10,006)	14.5% (1316)	15.5% (8690)
40–60	44.9% (29,225)	48.0% (4364)	44.4% (24,861)
60–80	37.1% (24,122)	34,7% (3153)	37.5% (20,969)
80+	2.6% (1675)	2.8% (257)	2.5% (1418)
Height, median (IQR)	172.0 (167.0–180.0)	170.0 (165.0–176.0)	173.0 (167.0–180.0)
Weight, median (IQR)	79.0 (68.0–90.0)	80.0 (69.0–93.5)	79.0 (68.0–90.0)
Body mass index (kg/m^2^), median (IQR)	26.0 (23.3–29.4)	27.3 (24.0–31.3)	25.8 (23.2–29.1)
Body mass index (kg/m^2^), % (*N*)			
0–25	41.1% (26,756)	32.8% (2978)	42.5% (23,778)
25–30	36.8% (23,924)	35.0% (3181)	37.1% (20,743)
30–35	15.1% (9838)	20.5% (1859)	14.3% (7979)
35–40	4.8% (3104)	7.6% (695)	4.3% (2409)
40+	2.2% (1406)	4.1% (377)	1.8% (1029)
Smoking status, % (*N*)			
Non‐smoker	70.0% (45,499)	62.2% (5657)	71.2% (39,842)
Previous smoker	21.7% (14,101)	25.4% (2313)	21.1% (11,788)
Smoker	8.3% (5428)	12.3% (1120)	7.7% (4308)
Civil status (living in a relationship), % (*N*)	39.1% (25,428)	42.1% (3827)	38.6% (21,601)
Accumulated income 2018–2020			
Value, median (IQR), 100.000	6.5 (4.8–8.4)	5.4 (4.1–7.0)	6.7 (5.0–8.6)
Missing, % (*N*)	0.4% (242)	0.3% (29)	0.4% (213)
Educational level, % (*N*)			
Low educational level	11.7% (7620)	18.6% (1690)	10.6% (5930)
Medium educational level	39.3% (25,544)	44.1% (4008)	38.5% (21,536)
High educational level	48.3 (31,435)	36.2% (3293)	50.3% (28,142)
Missing	0.7% (429)	1.1% (99)	0.6% (330)
Number of children living at home, median (IQR)	0.0 (0.0–1.0)	0.0 (0.0–1.0)	(0.0–1.0)
Number of children living at home (categorised), % (*N*)			
0	65.4% (42,550)	65.2% (5925)	65.5% (36,625)
1	13.7% (8930)	14.9% (1351)	13.5% (7579)
2	15.4% (10,034)	14.4% (1310)	15.6% (8724)
3+	5.4% (3514)	5.5% (504)	5.4% (3010)
Delay from first COVID questionnaire (months), % (*N*)			
8–11	54.6% (35,494)	55.0% (4998)	54.5% (30,496)
12–17	10.1% (6578)	10.9% (991)	10.0% (5587)
18–23	29.7% (19,290)	28.8% (2614)	29.8% (16,676)
24–32	5.6% (3666)	5.4% (487)	5.7% (3179)
Hospitalisation, % (*N*)			
Not hospitalised	95,5% (62,075)	92.1% (8371)	96.0% (53,704)
Hospitalised due to COVID‐19	3.8% (2498)	6.3% (576)	3.4% (1922)
Hospitalised not due to COVID‐19	0.5% (357)	1.2% (105)	0.5% (252)
Contracted COVID‐19 during hospitalisation	0.2% (98)	0.4% (38)	0.1% (60)

Abbreviations: IQR, inter‐quartile range; *N*, number.

### 3.1 Full Study Cohort (*n* = 65,028)

Participant data was collected on average 21.0 months (SD: 6.0 months) after the positive RT‐PCR test. A total of 14% reported pain before the SARS‐CoV‐2 infection. Table 1 describes in detail the socio‐demographics of three cohorts: (1) the full study cohort, (2) the pre‐COVID pain group, and (3) the non‐pre‐COVID pain group. Females were more prevalent (57.6%) and the percentage of females who reported pain before the infection (70.9% *n* = 6447) was higher compared to those who did not report pain (55.4%; *n* = 31,009). In the full study cohort, 17.7% reported post‐COVID pain. Compared to the non‐pre‐COVID pain group, the pre‐COVID pain group post‐COVID pain prevalence was higher (33.7% vs. 15.1%). Basic descriptive demographics were mostly similar in each subgroup compared to the full study cohort. Median age was 56.4 (46.2–65.6) in the full study cohort, 56.5 (46.2–65.9), and 55.9 (46.1–64.4) for the non‐pre‐COVID pain group, respectively. When stratifying into age brackets, age 40–60 is more prevalent in the pre‐COVID pain group (48.0% compared to 44.9% for the full study cohort). Median Body Mass Index (BMI) was 27.3 (24.0–31.3) in the pre‐COVID pain group, which was a little higher than in the full study cohort (Median 26.0; 23.3–29.4). The pre‐COVID pain group included more smokers (12.3%) and previous smokers (25.4%) than the full study cohort (8.3% and 21.7%, respectively).

Table 1 depicts the socio‐economic variables. Compared to the full study cohort, the educational level was lower in the pre‐COVID pain group, with medium educational level being more prevalent (44.1% vs. 39.3%). In the full study cohort, 11.7% were registered with low educational levels compared to 18.6% in the pre‐COVID pain group, while 36.2% were registered with high educational levels compared to 48.3% in the full study cohort. The median accumulated 3‐year income, in hundred thousand, was lower for the pre‐COVID pain group (5.4; 4.1–7.0) compared to the non‐pre‐COVID pain group (6.5; 4.8–8.4). In the pre‐COVID pain group, 6.3% were hospitalised because of COVID‐19, while 3.8% of the full study cohort participants were hospitalised.

### 3.2 Previous Conditions

Except for the free‐text comorbidity variable where participants could report any pre‐COVID condition not directly mentioned in the questionnaire, the pre‐COVID pain group reports a higher prevalence of pre‐COVID conditions, as seen in Table 2. The most prominent medical comorbidities for the pre‐COVID pain and non‐pre‐COVID pain groups were hypertension (25.0% and 19.7%) stress (11.5% and 5.1%), depression (11.4% and 4.6%), asthma (11.0% and 7.0%), and chronic neurological disease (10.3% and 1.4%).

**TABLE 2:** Medical conditions diagnosed pre‐COVID by a medical doctor stratified into the full study cohort, the pre‐COVID pain group, and the non‐pre‐COVID pain group.

	Full study cohort (*n* = 65.028)	Pre‐COVID pain group (*n* = 9090)	Non‐pre‐COVID pain group (*n* = 55,938)
Depression, % (*N*)	5.6% (3623)	11.4% (1035)	4.6% (2588)
Stress, % (*N*)	6.0% (3890)	11.5% (1041)	5.1% (2849)
Anxiety, % (*N*)	4.5% (2951)	9.4% (857)	3.7% (2094)
Type‐1 diabetes mellitus, % (*N*)	0.6% (368)	0.9% (84)	0.5% (284)
Type‐2 diabetes mellitus, % (*N*)	4.9% (3179)	7.2% (650)	4.5% (2529)
Asthma, % (*N*)	7.6% (4.914)	11.0% (1000)	7.0% (3914)
Dementia, % (*N*)	0.1% (97)	0.4% (34)	0.1% (63)
Cardiovascular disease, % (*N*)	5.1% (3298)	6.2% (565)	4.9% (2733)
Hypertension, % (*N*)	20.4% (13,291)	25.0% (2270)	19.7% (11,021)
Chronic obstructive lung disease, % (*N*)	1.9% (1209)	3.4% (310)	1.6% (899)
Chronic kidney disease, % (*N*)	0.5% (318)	0.8% (75)	0.4% (243)
Mild liver disease, % (*N*)	0.3% (179)	0.6% (50)	0.2% (129)
Non‐mild liver disease, % (*N*)	0.1% (81)	0.4% (32)	0.1% (49)
Asplenia, % (*N*)	0.0% (9)	*	*
Malicious tumour, % (*N*)	1.5% (969)	2.0% (186)	1.4% (783)
Post‐operative syndrome due to ICU admission, % (*N*)	0.0% (28)	0.1% (10)	0.0% (18)
Chronic neurological disease, % (*N*)	2.7% (1727)	10.3% (936)	1.4% (783)
Presence of comorbidities other than the above, % (*N*)	59.1% (38,436)	41.8% (3797)	61.9% (34,639)

Abbreviations: *, anonymized because of micro data < 3; *N*, number.

Of the 33.7% reporting pre‐COVID pain, 29.4% also reported using pain medication for their pain, see Table 3. In the full study cohort, this amounts to 4.1% using pain medication for their pain in general. Table 4 depicts the types of pre‐COVID pain reported by the participants in the pre‐COVID pain group. When asked about types of pain, the most prevalent pre‐COVID pain symptoms reported were back pain (42.8%), osteoarthritis (30.0%), shoulder and neck pain (28.0%), joint pain (24.3%), and muscle pain (22.3%).

**TABLE 3:** Overview of pain medication intake for the participants who reported one or more pre‐COVID pain symptoms stratified into the full study cohort, the pre‐COVID pain group, and the non‐pre‐COVID pain group.

	Full study cohort (*n* = 65.028)	Pre‐COVID pain group (*n* = 9090)	Non‐pre‐COVID pain group (*n* = 55,938)
Previous pain with pain medication, % (*N*)	4.1% (2669)	29.4% (2669)	100% (55,938)
Previous pain without pain medication, % (*N*)	9.9% (6421)	70.6% (6421)	0% (0)
No previous pain, % (*N*)	86.0% (55,938)	0% (0)	0% (0)

Abbreviation: *N*, number.

**TABLE 4:** Prevalence of pre‐COVID pain types reported by the participants stratified into the full study cohort, the pre‐COVID pain group, and the non‐pre‐COVID pain group.

	Full study cohort (*n* = 65.028)	Pre‐COVID pain group (*n* = 9090)	Non‐pre‐COVID pain group (*n* = 55,938)
Migraine, % (*N*)	1.8% (1162)	12.8% (1162)	0.0% (0)
Headache other than migraine, % (*N*)	1.7% (1089)	12.0% (1089)	0.0% (0)
Sore throat, % (*N*)	0.5% (334)	3.7% (334)	0.0% (0)
Breathing pain, % (*N*)	0.5% (307)	3.4% (307)	0.0% (0)
Arthritis, % (*N*)	2.3% (1466)	16.1% (1466)	0.0% (0)
Osteoarthritis, % (*N*)	4.2% (2731)	30.0% (2731)	0.0% (0)
Back pain, % (*N*)	6.0% (3886)	42.8% (3886)	0.0% (0)
Stomach pain, % (*N*)	1.0% (623)	6.9% (623)	0.0% (0)
Shoulder or neck pain, % (*N*)	3.9% (2544)	28.0% (2544)	0.0% (0)
Breast pain, % (*N*)	0.5% (334)	3.7% (334)	0.0% (0)
Whiplash, % (*N*)	1.1% (686)	7.5% (686)	0.0% (0)
Neuropathic pain, % (*N*)	1.6% (1047)	11.5% (1047)	0.0% (0)
Other nerve disease, % (*N*)	0.4% (277)	3.0% (277)	0.0% (0)
Postoperative pain, % (*N*)	0.8% (519)	5.7% (519)	0.0% (0)
Joint pain, % (*N*)	3.4% (2205)	24.3% (2205)	0.0% (0)
Muscle pain, % (*N*)	3.1% (2025)	22.3% (2025)	0.0% (0)
Presence of pain other than the above, % (*N*)	2.7% (1745)	19.2% (1745)	0.0% (0)

Abbreviation: *N*, number.

### 3.3 Predictive Ability for the Presentation of General Post‐COVID Pain of the Predictors

**FIGURE 2:**
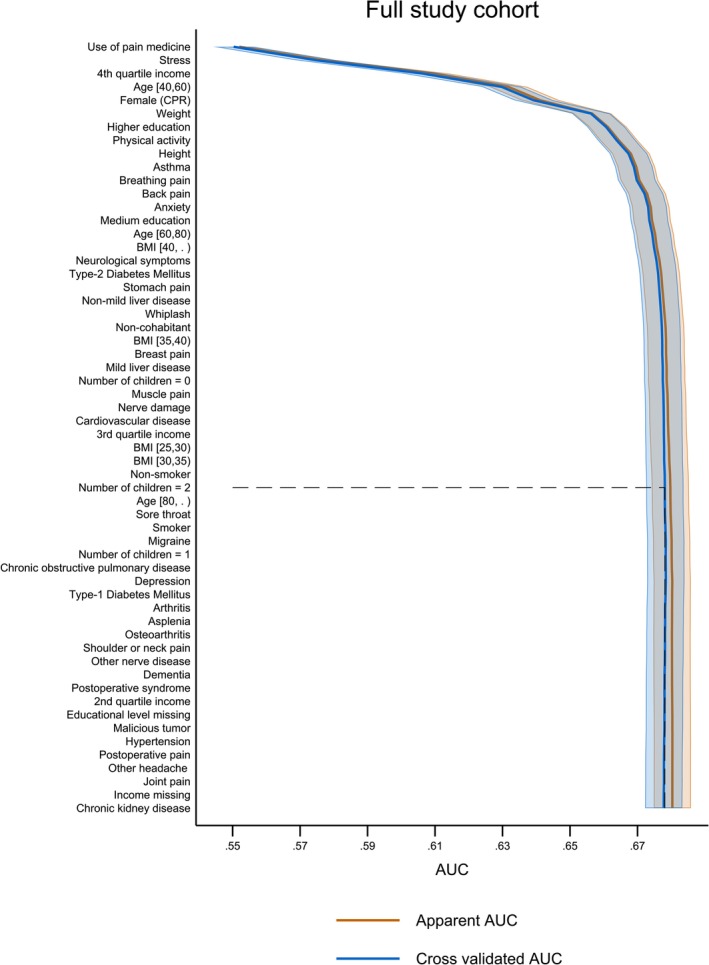
Apparent (blue lines) and cross‐validated (orange lines) Area under the Curve depicting the performance of Model 1 of the full study cohort and the complete list of included predictors ranked by the level of contribution to the predictive ability of Model 1. The dotted line is the model cut‐off point. The primary population performance included 34 predictors before cut‐off.

*Full study cohort Model* 1: The forward‐selected logistic regression model on the full study cohort of 65,028 participants that completed the questionnaires had a 5‐fold cvAUC of 0.68 (CI 0.67; 0.68). Of the 58 predictors available, the forward‐selection stopping process terminated at 34 predictors. In Table 5, the order of the included predictors is listed. The predictors included in Model 1 were then reported (Table 5). The multivariate apparent AUCs and cvAUCs at each step of the forward selection are shown graphically in Figure 2.

**TABLE 5:** Ranked contribution of the self‐reported pre‐COVID diagnosis predictors accepted by the model before reaching the cutoff point for Models 1‐3 of the full study cohort, the pre‐COVID pain group, and the non‐pre‐COVID pain group, respectively.

Rank	Full study cohort (*n* = 65.028)	Pre‐COVID pain group (*n* = 9090)	Non‐pre‐COVID pain group (*n* = 55,938)
1	Use of pain medication	Use of pain medication	Stress
2	Stress	Breathing pain	Female (CPR)
3	4th quartile income	Stress	Weight
4	Age [40,60]	Height	Higher education
5	Female (CPR)	Physical activity	Age [40,60]
6	Weight	Weight	4th quartile income
7	Higher education	Age [60,80]	Physical activity
8	Physical activity	Stomach pain	Height
9	Height	Type‐2 diabetes mellitus	Asthma
10	Asthma	Breast pain	Neurological symptoms
11	Breathing pain	4th quartile income	Anxiety
12	Back pain	Whiplash	Medium education
13	Anxiety	Mild liver disease	Age [60,80]
14	Medium education	Anxiety	BMI (kg/m^2^) [40+]
15	Age [60,80]	BMI (kg/m^2^) [30,35]	Non‐smoker
16	BMI (kg/m^2^) [40+]	Arthritis	BMI (kg/m^2^) [25,30]
17	Neurological symptoms	Osteoarthritis	Non‐cohabitant
18	Type‐2 Diabetes Mellitus	Muscle pain	Non‐mild liver disease
19	Stomach pain	Nerve damage	Cardiovascular disease
20	Non‐mild liver disease	Higher education	BMI (kg/m^2^) [30,35]
21	Whiplash	Medium education	Depression
22	Non‐cohabitant	Non‐mild liver disease	Type‐2 diabetes mellitus
23	BMI (kg/m^2^) [35,40]	Smoker	Smoker
24	Breast pain	Asthma	Not applicable
25	Mild liver disease	Not applicable	Not applicable
26	Number of children = 0	Not applicable	Not applicable
27	Muscle pain	Not applicable	Not applicable
28	Nerve damage	Not applicable	Not applicable
29	Cardiovascular disease	Not applicable	Not applicable
30	3rd quartile income	Not applicable	Not applicable
31	BMI (kg/m^2^) [25,30]	Not applicable	Not applicable
32	BMI (kg/m^2^) [30,35]	Not applicable	Not applicable
33	Non‐smoker	Not applicable	Not applicable
34	Number of children = 2	Not applicable	Not applicable

Abbreviation: *N*, number.

The first six predictors for post‐COVID pain as an outcome, chosen by the forward selection, contributed to a cvAUC of 0.66 (CI 0.65; 0.66), equalling 97.0% of the Model 1 performance when all 34 predictors until cut‐off were included. Those predictors, including univariate AUC, were use of pain medication (0.55), stress (0.53), 4th quartile (highest versus lowest income quartile) income (0.55), age [40,60] (0.54), female (CPR) (0.56), and weight (0.53). The remaining 27 predictors before cut‐off amounted to an increase in performance of 3.0%. Of the 34 predictors included in Model 1, seven (use of pain medication, breathing pain, back pain, Stomach pain, whiplash, breast pain, muscle pain) were related to previous pain conditions. Pre‐COVID conditions included nine (stress, asthma, anxiety, neurological symptoms, type‐2 Diabetes Mellitus, non‐mild liver disease, mild liver disease, nerve damage, cardiovascular disease) predictors. Socio‐demographic and socio‐economic factors comprised 11 (age [40,60], female (CPR), weight, physical activity, height, age [60,80], BMI [40+], BMI [35,40], BMI [25,30], BMI [30,35], non‐smoker) and seven (4th quartile income, higher education, medium education, non‐cohabitant, number of children = 0, 3rd quartile income, number of children = 2) predictors, respectively. For a complete overview of the predictor selection for Model 1 with 58 predictors, please refer to Table S3.

### 3.4 Pre‐COVID Pain and Non‐Pre‐COVID Pain Groups Prediction Profile Differences

To investigate the effect of pre‐COVID pain on the outcome, two additional forward‐selected logistic regression models (Models 2 and 3) were constructed and applied to the pre‐COVID pain (*n* = 9090) and non‐pre‐COVID pain (*n* = 55,938) groups. In both models, the first predictors explained most of the predictive value.

*Pre‐COVID pain group Model* 2: On the pre‐COVID pain of 9090 participants, Model 2 achieved a 5‐fold cvAUC of 0.69 (CI 0.68; 0.70), when forwardly selecting 24 predictors until the first drop in cvAUC. Of those, the first six predictors resulted in a cvAUC of 0.68 (CI 0.66; 0.69) which equalled 98.6% of the model performance. The predictors were: use of pain medication (0.64), breathing pain (0.52), stress (0.53), height (0.54), physical activity (0.52), and weight (0.51). The remaining 18 predictors amounted to an increase of 1.4% in Model 2 performance. Table S4 contains an overview of all predictors in Model 2.

*Non‐pre‐COVID pain group Model* 3: Model 3 applied to the 55,938 participants without pre‐COVID pain achieved a 5‐fold cvAUC of 0.65 (CI 0.64; 0.65). Of the 23 predictors included in Model 3 until cut‐off, the first seven predictors contributed to a cvAUC of 0.64 (CI 0.63; 0.64), equalling 98.5% of the performance of Model 3 with 23 predictors. The seven predictors were: stress (0.53), female (CPR) (0.55), weight (0.54), higher education (0.54), age [40,60] (0.54), 4th quartile income (0.55), and physical activity (0.52). The remaining 17 predictors constituted an increase in Model 3 performance of 1.5%. Please refer to Table S5 for an overview of predictors in Model 3.

### 3.5 Predictors Overlap Between the Full Study Cohort, the Pre‐COVID and Non‐Pre‐COVID Pain Groups

When considering the predictors that contribute the most to the AUC of Models 1, 2, and 3, stress (full study cohort: 0.53, pre‐COVID pain group: 0.53, non‐pre‐COVID pain group: 0.53) and weight (full study cohort: 0.53, pre‐COVID pain group: OR 0.51, non‐pre‐COVID pain group: 0.54) were overlapping in all three groups. In addition, physical activity was overlapping in the pre‐COVID pain group (0.52) and the non‐pre‐COVID pain group (0.52). The three models also included unique predictors until cut‐off. The full study cohort model uniquely featured back pain (0.54), 3rd quartile income (0.50), and participants having zero (0.51) or two children (0.50). The pre‐COVID pain group model uniquely included arthritis (0.50) and osteoarthritis (0.51). Finally, the non‐pre‐COVID pain group model uniquely included depression (0.52).

### 3.6 Summary of Top Predictor Profiles

For the full study cohort model, the pre‐COVID pain model, and the non‐pre‐COVID pain model, the top‐ranked predictors' univariate AUC and stepwise cvAUC have been depicted in Table 6. Compared to the total model performances of 0.68, 0.69, and 0.65, respectively, the relative contributions to each model have been summed up between each step to show the increasing predictive value of the previously selected predictors.

**TABLE 6:** Summary table of the ranked top predictor profiles in Models 1‐3. The first six predictors constituted 97% of the total predictive ability of Model 1. The first six predictors of Model 2 contributed to 98.6%, and the 7 highest‐ranked predictors of Model 3 contributed to 98.5% of the predictive value of Model 3.

Rank	Models 1–3	Univariate AUC	Cross‐validated stepwise AUC	% of total model performance
Model 1: Full study cohort (*n* = 65,028)				
1	Use of pain medicine	0.55 (0.55; 0.56)	0.55 (0.54; 0.56)	80.9%
2	Stress	0.53 (0.53; 0.53)	0.57 (0.57; 0.58)	83.8%
3	4th quartile income	0.55 (0.55; 0.55)	0.61 (0.60; 0.61)	89.7%
4	Age [40,60]	0.54 (0.53; 0.54)	0.63 (0.62; 0.64)	92.6%
5	Female (CPR)	0.56 (0.55; 0.56)	0.64 (0.63; 0.64)	94.1%
6	Weight	0.53 (0.53; 0.54)	0.66 (0.65; 0.66)	97.0%
Model 2: Pre‐COVID pain group (*n* = 9090)				
1	Use of pain medicine	0.64 (0.63; 0.65)	0.63 (0.62; 0.64)	91.3%
2	Breathing pain	0.52 (0.51; 0.52)	0.64 (0.62; 0.65)	92.8%
3	Stress	0.53 (0.52; 0.53)	0.65 (0.63; 0.66)	94.2%
4	Height	0.54 (0.53; 0.55)	0.66 (0.65; 0.68)	95.7%
5	Physical activity	0.52 (0.51; 0.53)	0.67 (0.66; 0.68)	97.1%
6	Weight	0.51 (0.49; 0.52)	0.68 (0.66; 0.69)	98.6%
Model 3: Non‐pre‐COVID pain group (*n* = 55,938)				
1	Stress	0.53 (0.52; 0.53)	0.52 (0.51; 0.53)	80.0%
2	Female (CPR)	0.55 (0.55; 0.56)	0.57 (0.56; 0.57)	87.7%
3	Weight	0.54 (0.53; 0.55)	0.61 (0.60; 0.62)	93.8%
4	Higher education	0.54 (0.54; 0.55)	0.62 (0.61; 0.62)	95.4%
5	Age [40,60]	0.54 (0.53; 0.55)	0.62 (0.62; 0.63)	95.4%
6	4th quartile income	0.55 (0.54; 0.55)	0.63 (0.62; 0.64)	96.9%
7	Physical activity	0.52 (0.51; 0.52)	0.64 (0.63; 0.64)	98.5%

### 3.7 Visualisation of the Models

**FIGURE 3:**
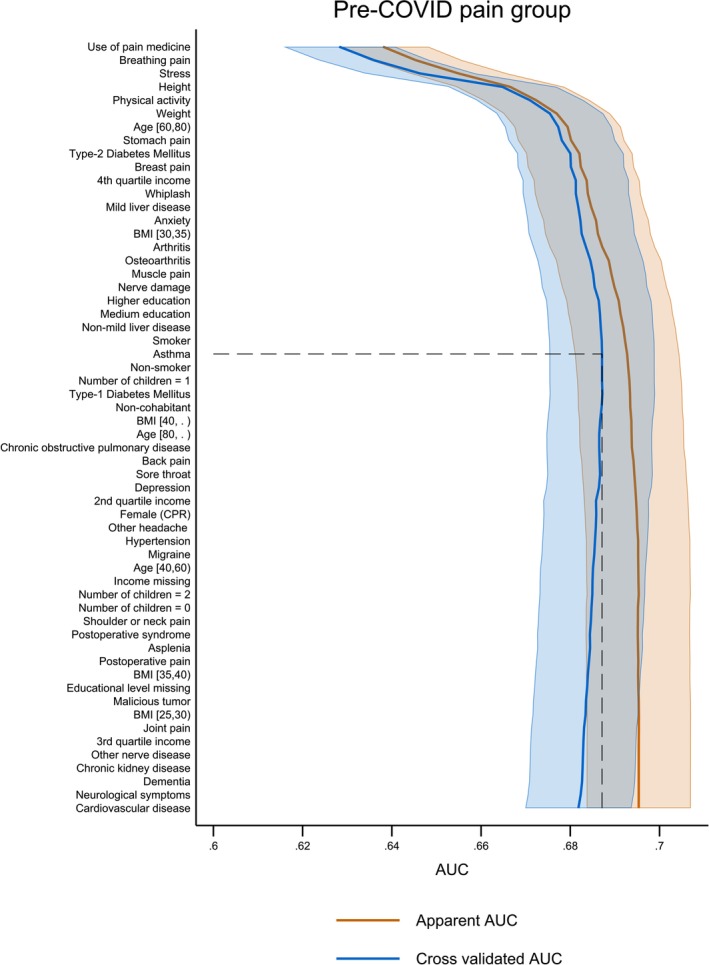
Apparent (blue lines) and cross‐validated (orange lines) Area under the Curve depicting the performance of Model 2 of the pre‐COVID pain group and the complete list of included predictors ranked by the level of contribution to the predictive ability of Model 2. The dotted line is the model cut‐off point. The pre‐COVID pain group performance included 24 predictors before cut‐off.

**FIGURE 4:**
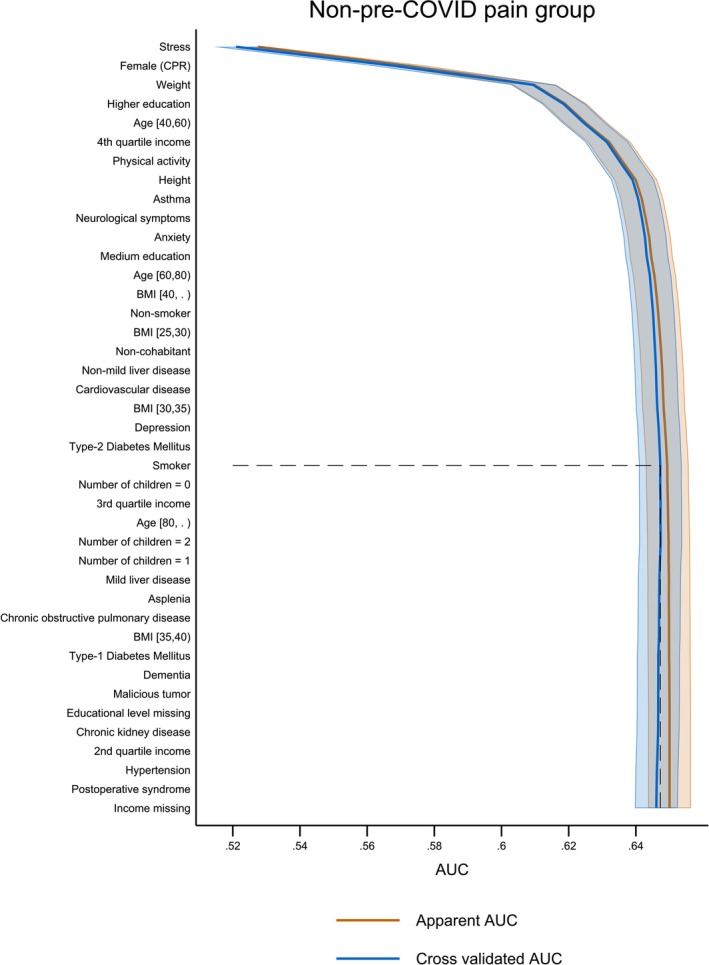
Apparent (blue lines) and cross‐validated (orange lines) Area under the Curve depicting the performance of Model 3 of the non‐pre‐COVID pain group and the complete list of included predictors ranked by the level of contribution to the predictive ability of Model 3. The dotted line is the model cut‐off point. The non‐pre‐COVID pain group performance included 23 predictors before cut‐off.

The performance of the three models was visualised in Figures 2, 3, 4 for the full study cohort, the pre‐COVID pain group, and the non‐pre‐COVID pain group, respectively. Figure 2 depicts the apparent and cross‐validated AUC for Model 1 featuring a small difference between the apparent AUC and the cvAUC curve throughout the included predictors with no significant decrease after model cut‐off (predictor 34: *number of children* = 2) suggesting that Model 1 was not subject to overfitting.

The pre‐COVID pain group cvAUC value (Figure 3) decreased significantly after Model 2 cut‐off (predictor 24: *asthma*). In general, the apparent AUC and cvAUC values were farther apart, which was likely due to a reduced sample size compared to the full study cohort and non‐pre‐COVID pain group and indicated a slightly larger likelihood of overfitting for the forward‐selected Model 2 containing 24 predictors.

The apparent and cvAUC curves, depicting Model 3 in Figure 4, decreased slightly after the model cut‐off (predictor 23: *smoker*). Only a small difference between the apparent and cvAUCs and a very small decrease in cvAUC were seen after cut‐off.

The Model 1–3 curves flattened with only marginal increases in predictive ability, reflected by changes in the third decimal or less, several steps back in the models before cut‐off where many predictors only contribute minimally to the predictive ability of the models.

To investigate the stopping criteria's robustness, we performed the analyses with 100 distinct cross‐validation splits to estimate the distribution of stopping points for the full study cohort and the pre‐COVID and non‐pre‐COVID pain groups. Figures [Link], [Link], [Link] depict the number of included predictors for each cross‐validation split for the full study cohort, the pre‐COVID pain group, and the non‐pre‐COVID pain group, respectively. There were some variations in the stopping criteria but in all cases, Models 1–3 included more predictors (ranging from 21 to 37 in the full study cohort and 9 to 27 and 18 to 28 in the pre‐COVID pain and non‐pre‐COVID pain groups, respectively). This shows that not too much emphasis should be placed on the exact stopping points, but rather on the number of variables that meaningfully impact the predictive ability of Models 1–3. The selection ordering was found using p‐values, a commonly used selection criterion. We also selected using AUCs for all three groups to analyse sensitivity and found a roughly similar ordering, suggesting that our results are not overly sensitive to the criterion choice for variable sequence selection. For the current data, only the first 6–7 predictors were emphasised as predictors of interest. Multicollinearity concerns about uncertainties of the predictor rankings were addressed by bootstrapping the distributions of individual variable rankings. For each of the three cohorts, 200 bootstrap samples were performed showing that each of the three cohorts had stable rankings, (see Figures [Link], [Link], [Link]). In summary, when looking at the cvAUCs in Figures S2–, S4, only a few of the included predictors made up the main predictive value. Here, stress and weight were common to all three groups. The use of pain medicine was common in the full study cohort and pre‐COVID pain group. Age [40,60], 4th quartile income, and female (CPR) were common in the full study cohort and non‐pre‐COVID pain group. Physical activity was common in the pain and non‐pre‐COVID pain groups.

**FIGURE 5:**
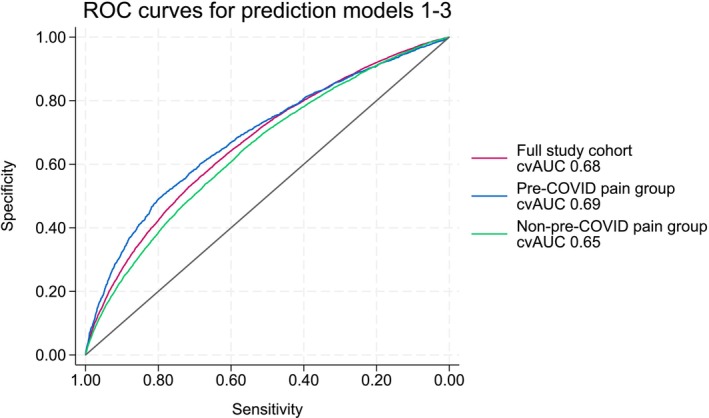
Apparent AUCROC curves showing the predictive value of Models 1–3: (in red) the full study cohort (*n* = 65,028), (in blue) the pre‐COVID pain group, and (in green) the non‐pre‐COVID pain group.

The performances of the three forward‐selected Models 1–3 were visualised in Figure 5 where the cross‐validated ROC curves of Models 1–3 are depicted. The red line represents Model 1 with a cvAUC of 0.68. The pain and non‐pre‐COVID pain groups in blue (cvAUC = 0.69) and green (cvAUC = 0.65) are depicted, respectively.

### 3.8 Implications of the Results

Table 1 highlighted the sociodemographic information collected about the participants stratified in the full study cohort, the pre‐COVID pain, and the non‐pre‐COVID pain groups. In particular, 17.7%, 33.7%, and 15.1% in the three groups respectively, reported post‐COVID pain. In Tables 2 and 4, the number of participants reporting pre‐COVID medical conditions and pre‐COVID pain conditions were shown. Table 3 elaborated on the use of pain medication for the participants reporting pre‐COVID pain conditions. For an overview of the predicted factors included in the final prediction models, Table 5 ranked the predictors stratified on the three groups for Models 1–3. Finally, Table 6 highlighted the 6, 6, and 7 predictors for Models 1–3, respectively, which constituted 97%–98.6% of the total performance of the models. Figures 2, 3, 4 showed the 58 (full study cohort and pre‐COVID pain group) and 41 (non‐pre‐COVID pain group) risk factors serving as input for the predictive models. In addition, the figures showed the cut‐off value of 34, 24, and 23 included predictors, respectively, in the final models. The cvAUCs were used as stopping criteria when the AUC would not increase. In‐depth numbers and rankings can be found in Tables S6–S8. In Figure 5, the specificity and sensitivity of Models 1–3 were summarised in ROC‐curves showing a cvAUC model performance of 0.68, 0.69, and 0.65, respectively. We have highlighted that only a few predictors provide most of the predictive ability of each model. Research‐wise, the implications of this were that emphasis should be placed on these predictors for further investigation. By focusing on these fewer predictors, this could lead to a more feasible clinical approach at a later stage in the research paradigm of predicting who could be at risk of developing post‐COVID pain.

## 4 Discussion

This study explored the predictive value of 58 potential pre‐COVID risk factors in a predictive model (Model 1, *n* = 65,028) and investigated potential differences in predictive risk factor profiles in people who had pre‐COVID pain (Model 2, *n* = 9090) and those who had no pre‐COVID pain (Model 3, *n* = 55,938). The prediction models were designed to investigate the predictive performance of potential predictors rather than which features (younger/older, higher/lower height and weight) were determining factors. The 3 models achieved 5‐fold cross‐validated AUCs ranging from 0.69 to 0.65. Of the 58 predictors, six contributed to 97% of Model 1 performance, 6 to 98.6% of Model 2, and 7 predictors to 98.5% of Model 3 performance. Age, weight, and stress were common predictors of interest across Models 1–3. This study highlighted important features of potential risk factors compared to previously identified risk factors, suggesting that certain predictors of interest should be the subject of further, more detailed investigation to help accommodate a more effective preventive effort against the risk of developing post‐COVID pain.

### 4.1 Full Study Cohort Features

The target population was the adult Danish part of the population who contracted COVID‐19 in Denmark until 31 December 2021. Considering the wide propagation of COVID‐19 among the general population (12.5% at the time of final inclusion (Statistics Denmark 2021)), the full study cohort was considered a heterogeneous sample of the target population in this period. There was an overrepresentation of females in the full study cohort, with a response rate of 57.6%. Females represented 70.9% of the pre‐COVID pain group. The target population consisted of 50.5% females, indicating that our sample was not strictly representative of COVID‐19‐infected adults. However, other large cohorts previously investigating COVID‐19 were considered comparable (Brannock et al. 2023; Hastie et al. 2023; Menni et al. 2022; Sørensen et al. 2022). A possible explanation for the difference in gender distribution might be that general response rates for males are lower (Wu et al. 2022), and because data was collected in two rounds of questionnaires, this might have skewed the gender distribution further. Finally, since females are more likely to suffer from long‐COVID, the gender distribution in the full study cohort was expected (Cohen and van der Meulen Rodgers 2023; Ebbesen, Giordano, Valera‐Calero, et al. 2024; Ebbesen, Giordano, Hedegaard, et al. 2024; Notarte et al. 2022; Subramanian et al. 2022).

### 4.2 Model Performance

Selecting a relatively simple model was advantageous in this explorative initial stage of post‐COVID pain research (Militino et al. 2024). The main purpose was to highlight variables containing predictive value in the full study cohort, the pre‐COVID, and non‐pre‐COVID pain groups. Also, forward‐selecting based on p‐values reduced the likelihood of introducing collinearity (Chowdhury and Turin 2020); hence, such forward selection tends to include predictors that contain information not already included in the previous step. This allows for the detection of fewer but more relevant predictors.

### 4.3 Contributing Predictors of Interest

The applied algorithm suggested a moderate predictive ability (Hosmer Jr. et al. 2013). Of 58 predictors selected for inclusion in Model 1, the six highest‐ranked predictors (Use of pain medicine, stress, 4th quartile income, age [40,60], female, and weight) contributed to a cvAUC of 0.66. Our previous research identified several potential post‐COVID pain experience risk factors (Ebbesen, Giordano, Valera‐Calero, et al. 2024; Ebbesen, Giordano, Hedegaard, et al. 2024). Of those, being female, older age, stress, and low‐income levels were supported by the current study and in agreement with former literature (Gevers‐Montoro et al. 2023; Goldhaber et al. 2022; Hastie et al. 2022; Subramanian et al. 2022; Thompson et al. 2022; Tsampasian et al. 2023; Wang et al. 2022). In addition, clinical pre‐COVID conditions already mentioned in the applied questionnaires are well‐documented risk factors (Bergmans et al. 2024; Fernández‐de‐las‐Peñas, De‐la‐Llave‐Rincón, et al. 2022; Fernández‐de‐las‐Peñas, Cancela‐Cilleruelo, et al. 2023; Kerzhner et al. 2024; Subramanian et al. 2022; Thompson et al. 2022; Tsampasian et al. 2023). The predictor contributing the most (with the lowest p‐value and high univariate AUC) was related to the severity of pain reported by participants in the full study cohort and the pre‐COVID pain group. The use of pain medicine is associated with a more severe incidence of pain in general (Hong et al. 2015; Pfaff et al. 2022; Shi et al. 2007) but is not well documented in the current literature concerning post‐COVID pain. In fact, with the post‐COVID pain phenotype potentially being nociplastic, and thus including a central component (Castaldo et al. 2023; Fernández‐de‐las‐Peñas, Nijs, et al. 2022), the use of pain medicine that normally targets peripheral phenomena might be less responsive (Fitzcharles et al. 2021). In turn, there might be an elevated number of participants reporting a medicinal intake. This could be a result of participants turning to pain medicine or the use of more pain medicine than previously to battle this new experienced pain phenotype. Weight, found to be an individual predictor for participants with post‐COVID pain regardless of pre‐COVID pain or not, was not considered a risk factor in our previous research. However, obesity (Abumweis et al. 2022; Subramanian et al. 2022; Thompson et al. 2022; Ursini et al. 2021) and higher BMI (Bliddal et al. 2021; Ebbesen, Giordano, Valera‐Calero, et al. 2024; Ebbesen, Giordano, Hedegaard, et al. 2024) have previously been linked to post‐COVID pain. Furthermore, obesity has been correlated with the development of a higher number of post‐COVID pain conditions and long‐COVID conditions in general (Abumweis et al. 2022; Fernández‐de‐las‐Peñas, Torres‐Macho, et al. 2021). This discrepancy between weight as a risk factor and a predictive factor may be explained by how data was investigated (Schooling and Jones 2018). With weight being highlighted as a predictive factor rather than a risk factor, this allows us to discriminate the results from any indications of a potential causal relationship between post‐COVID pain as an outcome and weight as a retrospective measurement of this outcome in the current cohort. The above‐mentioned predictors of interest added value they could not do as risk factors. They were not intended to explain the outcome. Rather, the predictors show an association of each predictor to post‐COVID pain as an outcome for possible identification of who might be at risk, thereby fostering hypothesis generation for future specific causal intervention research.

### 4.4 Pre‐COVID and Non‐pre‐COVID Pain Group Differences

*Use of pain medication and breathing pain*: Considering the use of pain medicine as a proxy for pain, this predictor represented pain in general and interpretation might be influenced by containing information about multiple pain predictors. However, recent literature showed that pre‐COVID pain was a driver of post‐COVID pain experience (Ebbesen, Giordano, Valera‐Calero, et al. 2024; Ebbesen, Giordano, Hedegaard, et al. 2024). In addition, respiratory and cardiovascular post‐COVID pain symptoms are among the most prominent COVID‐19 pain phenotypes (Davis et al. 2023b; Goldhaber et al. 2022; da Silva et al. 2022), supporting the Model 2 inclusion of use of pain medicine and breathing pain. Being modifiable predictors, this new knowledge could help direct attention towards the use of pain medicine and measures taken to reduce other pain conditions more systematically, knowing that this might reduce the risk of post‐COVID pain experience.

*Female gender*: Being female was considered less important (ranked 36), compared to the non‐pre‐COVID pain group (ranked as 2). Pain prevalence has been shown to be higher in women in general (Andrews et al. 2018), and the overrepresentation of women in the pre‐COVID pain group might have reduced the predictive value of gender.

*Height*: Height was considered more important in the pre‐COVID pain group (ranked 4 vs. 8). Women in general are shorter (Cavelaars et al. 2000). Thus, being female and height contain much of the same information in the stepwise progression of the model. Combined with a female overrepresentation, this may explain the difference between the pre‐COVID pain and non‐pre‐COVID pain groups.

*Higher income and education*: 4th quartile income was more important in the non‐pre‐COVID pain group (ranked 6 vs. 11) as was higher education (ranked 4 vs. 20). Previous literature already shows that social deprivation increases the risk of chronic pain (Prego‐Domínguez et al. 2021), long‐COVID in general (Shabnam et al. 2023; Subramanian et al. 2022), and post‐COVID pain (Buckley et al. 2023). It is possible that the differences in contribution to the performance of the predictive models can be ascribed to the higher prevalence of social deprivation in the pre‐COVID pain group. Low income and education and pain are correlated; thus, high income and education as predictors in the pre‐COVID pain group were less important, therefore ranking 4th income and higher education lower. Importantly, the modifiable features of social deprivation emphasise the need to understand the causal relationship to the risk of post‐COVID pain experience.

Generally, the between‐group differences in predictor inclusion and ranking were small in the full study cohort, the pre‐COVID and non‐pre‐COVID pain groups, suggesting that the six first variables chosen in Model 1 are important predictors of post‐COVID pain. Interestingly, with a cvAUC of 0.69, Model 2 showed higher predictive ability compared to the Model 3 performance of 0.65, supporting existing literature that pre‐COVID pain conditions increase the risk of post‐COVID pain (Castaldo et al. 2023; Fernández‐de‐las‐Peñas, De‐la‐Llave‐Rincón, et al. 2022; Goldhaber et al. 2022). Further, pre‐COVID multimorbidity in general and chronic overlapping pain conditions (COPCs) create interaction effects (Bergmans et al. 2024; Russell et al. 2023). These interaction effects might have been harder to detect in the pre‐COVID pain group encompassing more pain variables. A different approach that could investigate these interaction effects would be of interest to explore further. In addition, the pain‐related predictors included in Model 2 show that pre‐COVID pain might be an important predictor for the experience of post‐COVID pain (Ebbesen, Giordano, Valera‐Calero, et al. 2024, Ebbesen, Giordano, Hedegaard, et al. 2024) even when compared to a large non‐pain population.

### 4.5 Modifiable and Non‐Modifiable Predictors in General

In Models 1–3, the predictors have different properties, with some being non‐modifiable (Age, gender, and height) and the rest being factors that possibly can be modified. Some non‐modifiable factors might outweigh the benefits that can be achieved by modifying other predictors, such as income, education, and physical activity. Being female and the effect of age are factors that highly influence the prevalence of post‐COVID pain. Some research, however, suggests that age as an immutable factor might actually be deconstructed into time‐related events that in different ways can be affected (Sniderman and Furberg 2022). On the other hand, non‐modifiable predictors may be early signs of the potential for experiencing long COVID and post‐COVID pain (Huang et al. 2022). This is important knowledge if we want to understand not only what potential predictors of post‐COVID pain are but also how to act on this knowledge when treating people at risk of post‐COVID pain. To be able to understand how to act on potential post‐COVID pain predictors that are modifiable, future research must direct attention towards the specific predictors derived from the current study to investigate the correlation between those modifiable and non‐modifiable predictors and post‐COVID pain experience (Kostoff et al. 2023).

### 4.6 Limitations

Several limitations must be considered when prioritising the predictors in Models 1–3 for the current study. The self‐reported data used as the primary source was subject to potential biases mostly covered in our previous work (Ebbesen, Giordano, Valera‐Calero, et al. 2024, Ebbesen, Giordano, Hedegaard, et al. 2024) but response bias could result in misclassification due to misunderstandings or misinterpretations, for example, that reported pain could be a result of other events than a COVID‐19 infection. Also, non‐responder bias might influence the model performance. Secondly, being explorative, Models 1–3 were not constructed for application to other populations; thus, no external model validation was performed. Instead, the predictors of interest from the current study should inspire future research when investigating the predictive ability of risk factors for post‐COVID pain. No single type of model can provide a complete picture (Ethgen and Standaert 2012). The conservative approach applied to this study, to avoid overfitting and enhance transparency in the interpretation of results (Chollet Ramampiandra et al. 2023), may not capture the effects of multimorbidity or the complex inter‐variable relationships (Russell et al. 2023). Importantly, while previous research on risk factors focused on features of these, this model investigated the predictive ability of the selected predictors. Thus, a direct correlation between previously investigated risk factors and the predictors of this study should not be made. In addition, when talking about the prevalence of post‐COVID pain, the results are limited by not controlling for prevalence in non‐COVID populations. Previous research has shown how COVID‐19 sequelae prevalence, when controlled for non‐COVID populations, is lower (Sørensen et al. 2022). Thus, the prevalence used to construct the models in the current study might reflect a symptomatology also present outside of COVID‐19 survivors. Future research should direct attention to the clinical aspects of these predictors. Finally, the variable ranking generated in the main analysis is not necessarily true for everyone in the Danish population, as apparent from the subgroup analysis, where another prioritisation seems to be true.

## 5 Conclusion

This large‐scale cohort study of 65,028 Danish COVID‐19 survivors utilised self‐reported questionnaires and registry data to identify potential predictors for the presentation of post‐COVID pain. The prediction models identified the predictive performance of predictors not discriminating on distinctive features (higher/lower age, weight, and height as well as whether the individual predictors showed protective features). Model 1, incorporating 58 potential predictors to the full study cohort, identified the use of pain medicine, stress, 4th quartile (highest income versus the lowest quartile income) income, age [40,60], female gender, and weight as predictors of interest. For Models 2 and 3, based on the pre‐COVID pain and non‐pre‐COVID pain subgroups, we identified 6 (cvAUC = 0.69) and 7 (cvAUC = 0.65) potential predictors, respectively. Model 2 identified the use of pain medicine, breathing pain, stress, height, physical activity, and weight, and Model 3 identified stress, female gender, weight, higher education, age [40,60], highest income quartile, and physical activity. Of the predictors of interest in Models 1–3, the authors suggest investigating in more detail those that are modifiable. Predictors related to physical and mental health and social deprivation should be investigated for causal elements of the individual modifiable predictors to enable a more actionable approach in the prevention of post‐COVID pain development in future COVID‐19 survivors. In addition, the study might provide generic information that could apply to other serious viral infections susceptible to long‐term sequelae.

## Conflicts of Interest

The authors declare no conflicts of interest.

## Supporting information


**Figure S1.** Sensitivity analysis of the robustness of the stopping criteria in the full study cohort model. Cross‐validation splits were reperformed 100 times. The inclusion of predictors was within the distribution of the cross‐validation splits.

**Figure S2.** Sensitivity analysis of the robustness of the stopping criteria in the pre‐COVID pain group model. Cross‐validation splits were reperformed 100 times. The inclusion of predictors was within the distribution of the cross‐validation splits.

**Figure S3.** Sensitivity analysis of the robustness of the stopping criteria in the non‐pre‐COVID pain group model. Cross‐validation splits were reperformed 100 times. The inclusion of predictors was within the distribution of the cross‐validation splits.

**Figure S4.** Boxplot of 200 bootstraps for the full study cohort showing the distribution of the ranking of each predictor highlighting the uncertainty of the individual ranges in the main results supporting the only minor range uncertainty in the predictors of interest.

**Figure S5.** Boxplot of 200 bootstraps for the pre‐COVID pain group showing the distribution of the ranking of each predictor highlighting the uncertainty of the individual ranges in the main results supporting the only minor range uncertainty in the predictors of interest.

**Figure S6.** Boxplot of 200 bootstraps for the non‐pre‐COVID pain group showing the distribution of the ranking of each predictor highlighting the uncertainty of the individual ranges in the main results supporting the only minor range uncertainty in the predictors of interest.

Tables S1–S8.

Data S1.

## Data Availability

The anonymized questionnaire data sets are available upon request to the corresponding author. Due to the legal limitations applied by the Danish Health Act, § 42, subsection 1, supporting registered data is not available.
