# Supplementary figures and images for: Predictive Ability of Previous Pain and Disease Conditions on the Presentation of Post‐COVID Pain in a Danish Cohort of Adult COVID‐19 Survivors
Source: Eur J Pain. 2025 Apr 5;29(5):e70021. doi: 10.1002/ejp.70021 (PMC11971649; doi:10.1002/ejp.70021)

## Full study cohort

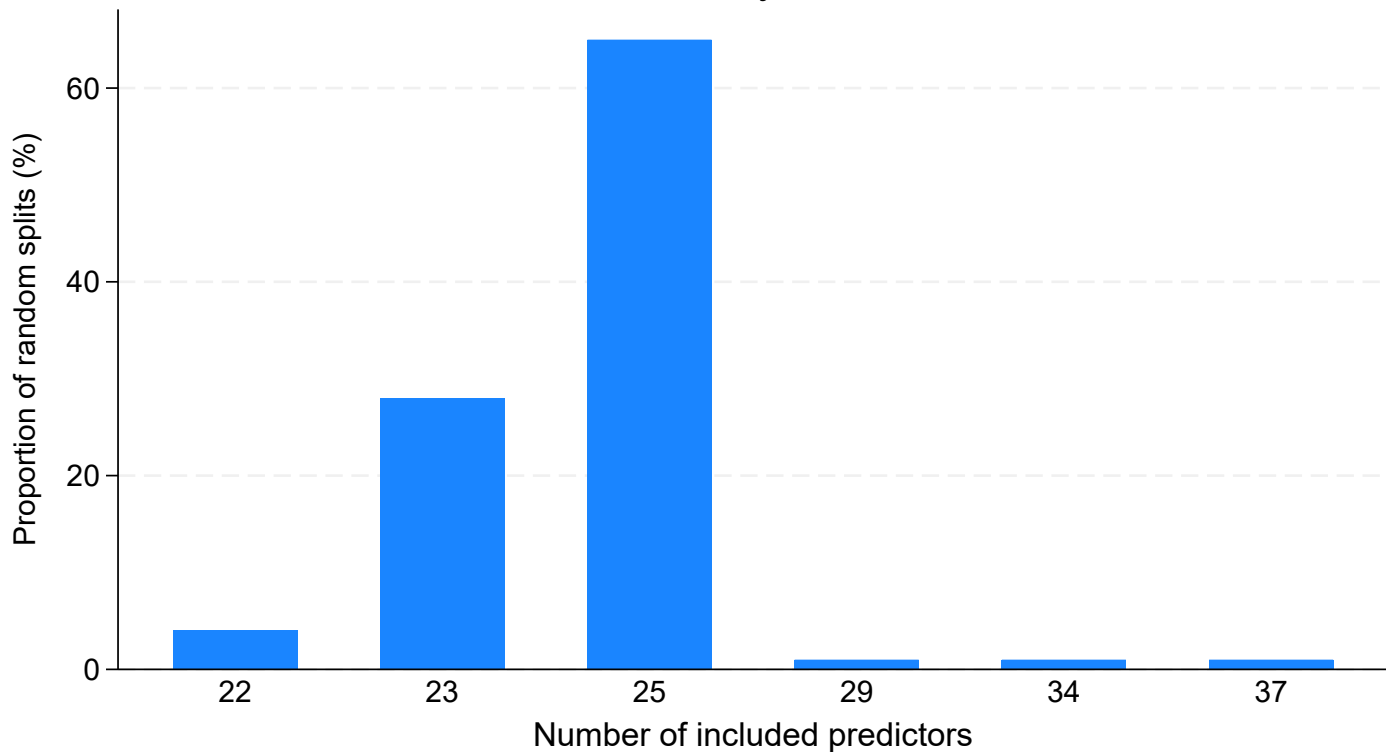

Supplement: Supplementary file 1 — Figure S1. Sensitivity analysis of the robustness of the stopping criteria in the full study cohort model. Cross‐validation splits were reperformed 100 times. The inclusion of predictors was within the distribution of the cross‐validation splits. [file EJP-29-0-s007.pdf]

## Pre-COVID pain group

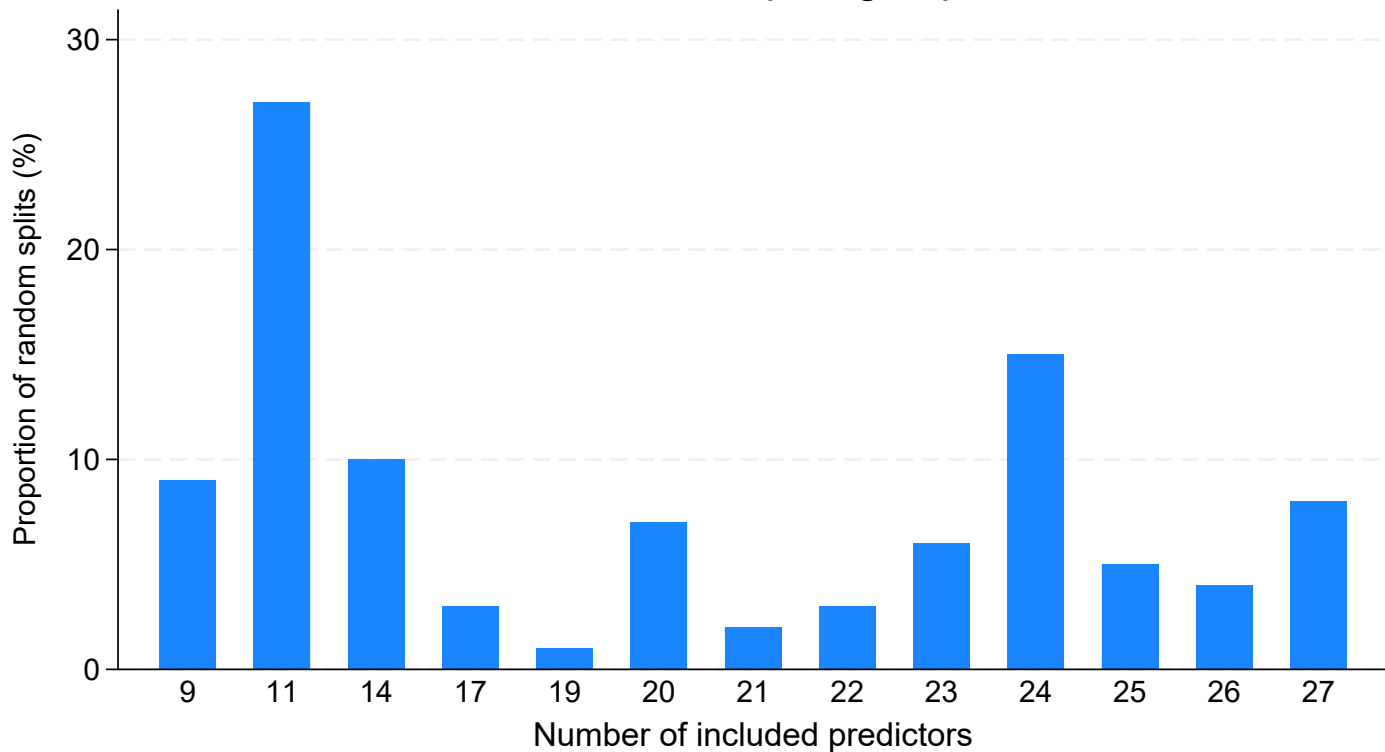

Supplement: Supplementary file 2 — Figure S2. Sensitivity analysis of the robustness of the stopping criteria in the pre‐COVID pain group model. Cross‐validation splits were reperformed 100 times. The inclusion of predictors was within the distribution of the cross‐validation splits. [file EJP-29-0-s004.pdf]

## Non-pre-COVID pain group

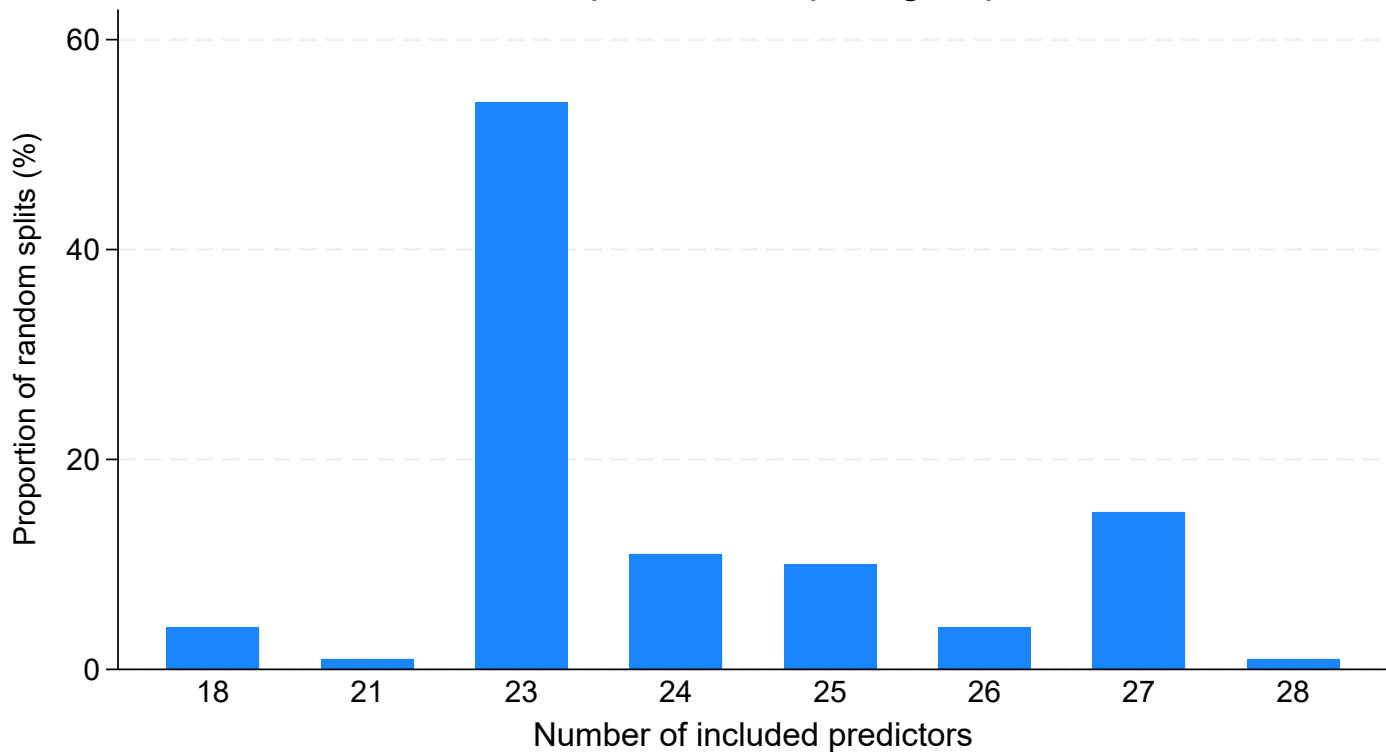

Supplement: Supplementary file 3 — Figure S3. Sensitivity analysis of the robustness of the stopping criteria in the non‐pre‐COVID pain group model. Cross‐validation splits were reperformed 100 times. The inclusion of predictors was within the distribution of the cross‐validation splits. [file EJP-29-0-s003.pdf]

# Full study cohort

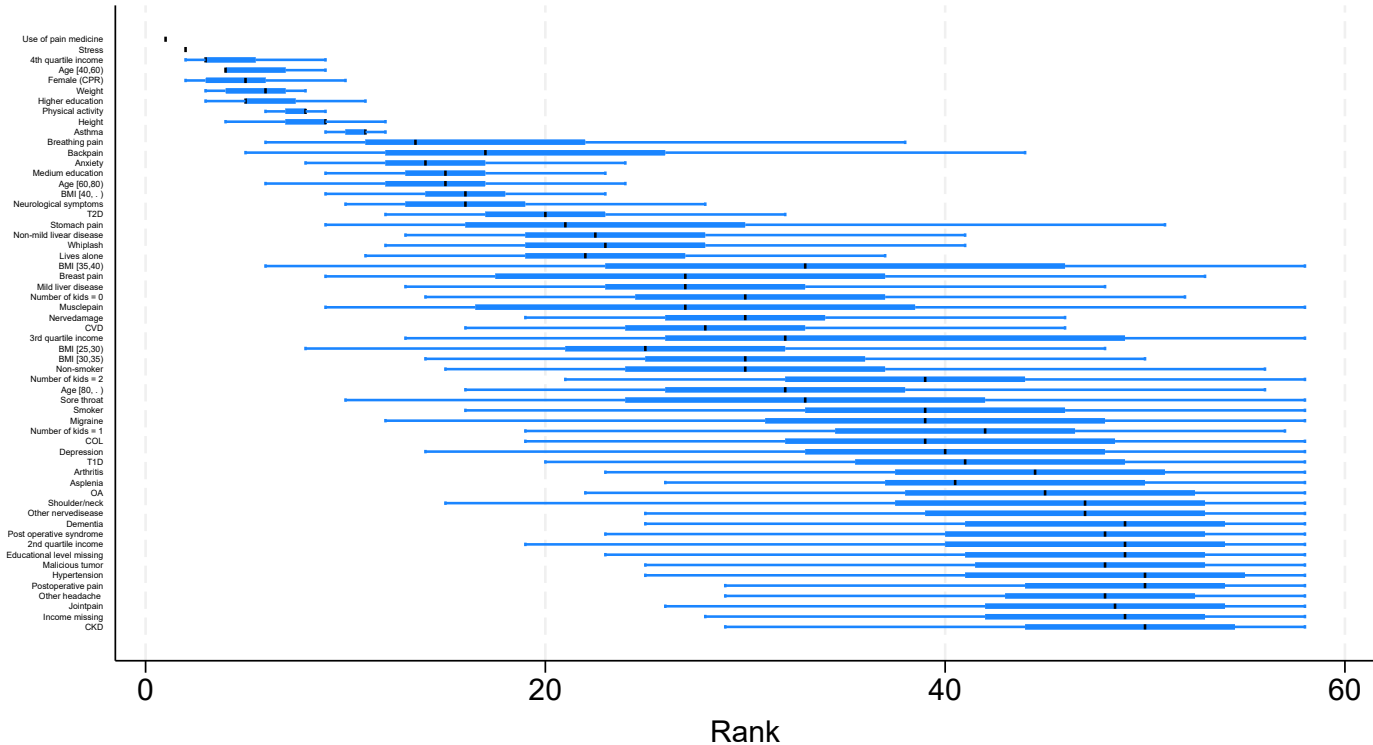

Supplement: Supplementary file 4 — Figure S4. Boxplot of 200 bootstraps for the full study cohort showing the distribution of the ranking of each predictor highlighting the uncertainty of the individual ranges in the main results supporting the only minor range uncertainty in the predictors of interest. [file EJP-29-0-s002.pdf]

# Pre-COVID pain group

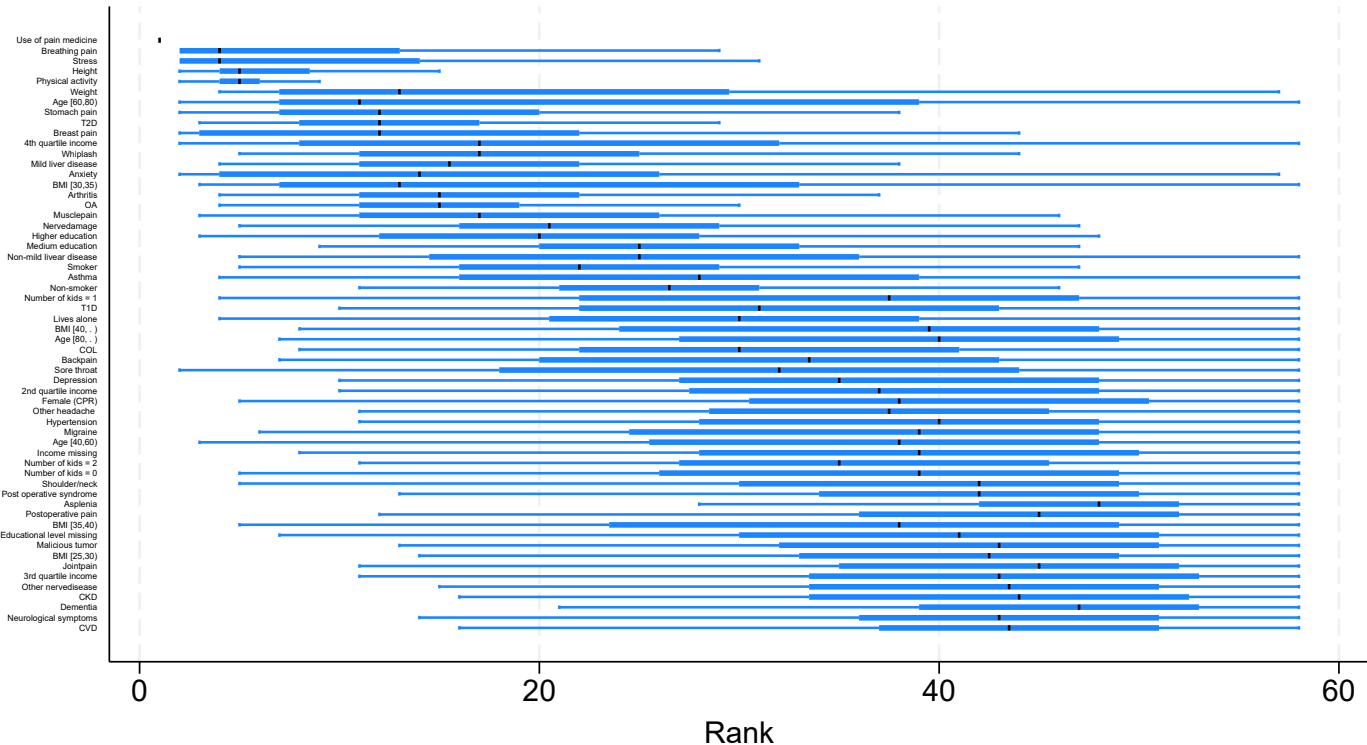

Supplement: Supplementary file 5 — Figure S5. Boxplot of 200 bootstraps for the pre‐COVID pain group showing the distribution of the ranking of each predictor highlighting the uncertainty of the individual ranges in the main results supporting the only minor range uncertainty in the predictors of interest. [file EJP-29-0-s008.pdf]

# Non-pre-COVID pain group

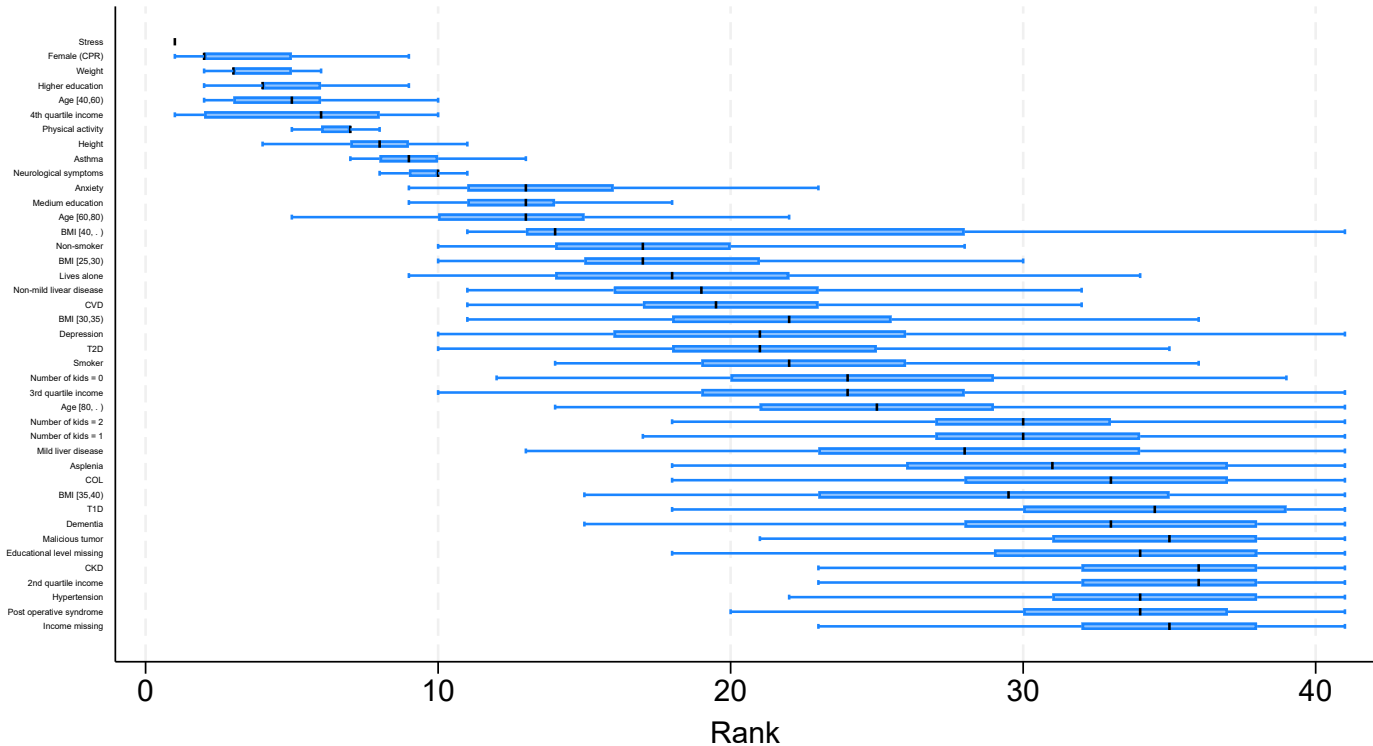

Supplement: Supplementary file 6 — Figure S6. Boxplot of 200 bootstraps for the non‐pre‐COVID pain group showing the distribution of the ranking of each predictor highlighting the uncertainty of the individual ranges in the main results supporting the only minor range uncertainty in the predictors of interest. [file EJP-29-0-s006.pdf]
