# Supplementary material for: Predictive Ability of Previous Pain and Disease Conditions on the Presentation of Post‐COVID Pain in a Danish Cohort of Adult COVID‐19 Survivors
Source: Eur J Pain. 2025 Apr 5;29(5):e70021. doi: 10.1002/ejp.70021 (PMC11971649; doi:10.1002/ejp.70021)
Supplement: Supplementary file 7 — Tables S1–S8. [file EJP-29-0-s005.docx]

**Table s1**: An overview of the predictor variables and their coding from the questionnaires and registered data. Where there is nothing under the category ‘Use’, the variable has been implemented directly.

| **Variable** | **Source** | **Definition** | **Use** |
| --- | --- | --- | --- |
| **De novo pain** | **Questionnaire** | **Either s_13 or s_14 marked** | **Outcome** |
| Sex (Female) | CPR | Sex assigned at birth or legal  gender if changed after birth | - |
| Age at the issue of  the first questionnaire | CPR | The difference in days between the  date of birth and 09/23/2022 | Binned according to breakpoints 40,60, and 80. |
| Accumulated income | INDAKK | Income accumulated between 2020 and 2018 | Binned according to quartiles |
| Educational level | AUDD | - | Binned into categories corresponding to ISCED levels (Low = 10 or 20, Medium = 30 or 40, High 50 or higher) |
| Number of  resident children | BEF | - | Binned according to breakpoints 1,2, and 3 |
| Non-cohabitant | BEF | Widowed, divorced, longest living of two partners, cancelled registered partnership, unmarried | Binary |
| Height | Questionnaire | s_3 | Continuous |
| Weight | Questionnaire | s_4 | Continuous |
| Body Mass Index (kg/m^2^) | Questionnaire | s_3, s_4 | Binned according to breakpoints 25,30,35, and 40 |
| Physical activity | Questionnaire | sn_2 | - |
| Smoking status | Questionnaire | sn_3 | All categories |
| Depression | Questionnaire | sn_4_1 | - |
| Stress | Questionnaire | sn_4_2 | - |
| Anxiety | Questionnaire | sn_4_3 | - |
| Type-1 Diabetes Mellitus | Questionnaire | sn_4_4 | - |
| Type-2 Diabetes Mellitus | Questionnaire | sn_4_5 | - |
| Asthma | Questionnaire | sn_4_6 | - |
| Dementia | Questionnaire | sn_4_7 | - |
| Cardiovascular disease | Questionnaire | sn_4_8 | - |
| Hypertension | Questionnaire | sn_4_9 | - |
| Chronic obstructive  lung disease | Questionnaire | sn_4_10 | - |
| Chronic kidney disease | Questionnaire | sn_4_11 | - |
| Mild liver disease | Questionnaire | sn_4_12 | - |
| Moderate to severe  liver disease | Questionnaire | sn_4_13 | - |
| Asplenia | Questionnaire | sn_4_14 | - |
| Malicious tumor | Questionnaire | sn_4_15 | - |
| Postoperative syndrome due to ICU admission | Questionnaire | sn_4_16 | - |
| Chronic  neurological disease | Questionnaire | sn_4_17 | - |
| None of the above | Questionnaire | sn_4_18 | Descriptive |
| Hospitalization | Questionnaire | s**_**5 | - |
| Pain prior to infection | Questionnaire | Defined as pain without medicine if s_7 = 1, as pain with the use of medicine if s_11 = 1, and as no pain otherwise. | - |
| Migraine | Questionnaire | s_8_1 | - |
| Other headache | Questionnaire | s_8_2 | - |
| Sore throat | Questionnaire | s_8_3 | - |
| Breathing pain | Questionnaire | s_8_4 | - |
| Arthritis | Questionnaire | s_8_5 | - |
| Osteoarthritis | Questionnaire | s_8_6 | - |
| Backpain | Questionnaire | s_8_7 | - |
| Stomach pain | Questionnaire | s_8_8 | - |
| Shoulder or neck pain | Questionnaire | s_8_9 | - |
| Breast pain | Questionnaire | s_8_10 | - |
| Whiplash | Questionnaire | s_8_11 | - |
| Nerve pain | Questionnaire | s_8_12 | - |
| Other nerve disease | Questionnaire | s_8_13 | - |
| Postoperative pain | Questionnaire | s_8_14 | - |
| Joint pain | Questionnaire | s_8_15 | - |
| Muscle pain | Questionnaire | s_8_16 | - |
| Other pain | Questionnaire | If anything was written in s_8_17 | Descriptive |
| Multi-type pain | Questionnaire | If two or more of s_8_1  to s_8_16 was marked | - |
| Number COVID-19 reinfections | Questionnaire | s_20 | Descriptive |
| Number of COVID-19 vaccines received | Questionnaire | s_24 | Descriptive |
| Not vaccinated | Questionnaire | s_26 | Descriptive |
| Delay from first COVID-19 infection to questionnaire data collection | Overvaagnings-data | Difference in days between the first positive COVID_19 RT-PCR test and 09/23/2022 | Descriptive |

**Table s2**: Overview of the questionnaire ID and labels of the question responses included in the analysis and coding for the prediction models 1-3.

| **Questionnaire ID** | **Questionnaire response** |
| --- | --- |
| s_2 | Age |
| s_3 | Height |
| s_4 | Weight |
| s_5 | Were you admitted to a hospital with Covid? |
| s_7 | Prior to you getting Covid, were you diagnosed by a medical doctor with any sort of long-term pain condition? |
| **s_8**  1  2  3  4  5  6  7  8  9  10  11  12  13  14  15  16  17 | **Which type(s) of long-term pain were you diagnosed with prior to you having COVID-19:**  Migraine  Any other types of headaches  Sore throat  Breathing pain  Arthritis  Osteoarthritis  Back pain  Abdominal pain  Shoulder or neck pain  Chest pain  Whiplash  Nerve damage  Other nerve disease  Postoperative pain  Joint pain  Muscle pain  Other pain than the above-mentioned |
| sn_2 | Prior to your Covid infection, were you physically active 30 minutes a day on average? |
| sn_3 | Prior to your Covid infection, were you smoking? |
| **sn_4**  1  2  3  4  5  6  7  8  9  10  11  12  13  14  15  16  17  18 | **Prior to your COVID-infection, were you diagnosed with one of the following diseases:**  Depression  Stress  Anxiety  Type-1 Diabetes Mellitus  Type-2 Diabetes Mellitus  Asthma  Dementia  Chronic cardiac disease  Hypertension  Chronic obstructive pulmonary disease  Chronic kidney disease  Mild liver disease  Moderate or severe liver disease  Asplenia  One or more malignant tumors  Post-intensive care syndrome because of previous admission to intensive care at a hospital  Chronic neurological disorder  None of the above-mentioned |
| s_11 | Do you take any medication for your pain? |
| s_13 | Have you developed pain after having had COVID-19? |
| s_14 | Have you developed new pain after having had COVID-19? |
| s_20 | How many times have you had COVID-19? |
| s_24 | Have you been vaccinated against COVID-19? (This includes booster injections you may have received) |
| s_26 | Relative to your vaccination(s), when were you infected with COVID-19? |

**Table s3**: Cross-validated forward stepwise prediction model of all predictors for the full study cohort (n=65,028). The predictors have been ranked by Model 1 and include predictors also ranked after the model cutoff. For each predictor, the values for the univariate and stepwise Area Under the Curve (AUC), the cross-validated stepwise values, the odds ratios from Model 1, and the univariate odds ratios are depicted.

|  | **Predictor** | **Univariate AUC** | **Stepwise**  **AUC** | **Cross-validated stepwise AUC** | **Model 1 odds ratio (CI)** | **Univariate odds**  **ratios (CI)** |
| --- | --- | --- | --- | --- | --- | --- |
| **1** | Use of pain medicine | 0.55 (0.55;0.56) | 0.55 (0.55;0.56) | 0.55 (0.54;0.56) | 3.90 (3.55;4.27) | 6.27 (5.79;6.78) |
| **2** | Stress | 0.53 (0.53;0.53) | 0.58 (0.57;0.58) | 0.57 (0.57;0.58) | 1.73 (1.60;1.88) | 2.46 (2.29;2.64) |
| **3** | 4^th^ quartile income | 0.55 (0.55;0.55) | 0.61 (0.60;0.61) | 0.61 (0.60;0.61) | 0.72 (0.68;0.77) | 0.55 (0.52;0.58) |
| **4** | Age [40,60] | 0.54 (0.53;0.54) | 0.63 (0.63;0.64) | 0.63 (0.62;0.64) | 1.23 (1.15;1.31) | 1.36 (1.30;1.41) |
| **5** | Female (CPR) | 0.56 (0.55;0.56) | 0.64 (0.64;0.65) | 0.64 (0.63;0.64) | 1.40 (1.32;1.50) | 1.62 (1.55;1.69) |
| **6** | Weight | 0.53 (0.53;0.54) | 0.66 (0.65;0.66) | 0.66 (0.65;0.66) | 1.01 (1.01;1.02) | 1.01 (1.01;1.01) |
| **7** | Higher education | 0.55 (0.54;0.55) | 0.66 (0.66;0.67) | 0.66 (0.66;0.67) | 0.70 (0.65;0.75) | 0.67 (0.65;0.70) |
| **8** | Physical activity | 0.51 (0.51;0.52) | 0.66 (0.66;0.67) | 0.66 (0.66;0.67) | 1.54 (1.45;1.65) | 1.24 (1.17;1.32) |
| **9** | Height | 0.56 (0.56;0.57) | 0.67 (0.66;0.67) | 0.67 (0.66;0.67) | 0.98 (0.98;0.99) | 0.98 (0.98;0.98) |
| **10** | Asthma | 0.52 (0.52;0.52) | 0.67 (0.66;0.68) | 0.67 (0.66;0.67) | 1.34 (1.24;1.44) | 1.61 (1.51;1.73) |
| **11** | Breathing pain | 0.51 (0.50;0.51) | 0.67 (0.66;0.68) | 0.67 (0.66;0.68) | 1.81 (1.37;2.40) | 5.75 (4.59;7.20) |
| **12** | Back pain | 0.54 (0.53;0.54) | 0.67 (0.67;0.68) | 0.67 (0.67;0.68) | 1.20 (1.09;1.30) | 2.79 (2.60;2.98) |
| **13** | Anxiety | 0.52 (0.52;0.52) | 0.67 (0.67;0.68) | 0.67 (0.67;0.68) | 1.28 (1.17;1.41) | 2.23 (2.06;2.42) |
| **14** | Medium education | 0.52 (0.52;0.53) | 0.67 (0.67;0.68) | 0.67 (0.67;0.68) | 0.85 (0.80;0.90) | 1.20 (1.16;1.25) |
| **15** | Age [60,80] | 0.53 (0.53;0.54) | 0.68 (0.67;0.68) | 0.67 (0.67;0.68) | 0.86 (0.80;0.92) | 0.74 (0.71;0.77) |
| **16** | BMI (kg/m^2^) [40, . ) | 0.51 (0.51;0.51) | 0.68 (0.67;0.68) | 0.67 (0.67;0.68) | 0.84 (0.64;1.12) | 1.94 (1.73;2.18) |
| **17** | Neurological symptoms | 0.51 (0.51;0.51) | 0.68 (0.67;0.68) | 0.68 (0.67;0.68) | 1.37 (1.22;1.55) | 2.16 (1.95;2.39) |
| **18** | Type-2 Diabetes Mellitus | 0.51 (0.51;0.51) | 0.68 (0.67;0.68) | 0.68 (0.67;0.68) | 1.21 (1.11;1.33) | 1.40 (1.29;1.52) |
| **19** | Stomach pain | 0.51 (0.51;0.51) | 0.68 (0.67;0.68) | 0.68 (0.67;0.68) | 1.39 (1.15;1.67) | 3.83 (3.27;4.49) |
| **20** | Non-mild liver disease | 0.50 (0.50;0.50) | 0.68 (0.67;0.68) | 0.68 (0.67;0.68) | 2.45 (1.51;3.97) | 3.72 (2.40;5.77) |
| **21** | Whiplash | 0.51 (0.51;0.51) | 0.68 (0.67;0.68) | 0.68 (0.67;0.68) | 1.38 (1.16;1.65) | 3.21 (2.76;3.75) |
| **22** | Non-cohabitant | 0.52 (0.52;0.53) | 0.68 (0.67;0.68) | 0.68 (0.67;0.68) | 1.09 (1.04;1.14) | 1.19 (1.15;1.24) |
| **23** | BMI (kg/m^2^) [35,40] | 0.51 (0.51;0.52) | 0.68 (0.67;0.68) | 0.68 (0.67;0.68) | 1.08 (0.90;1.31) | 1.74 (1.60;1.89) |
| **24** | Breast pain | 0.51 (0.51;0.51) | 0.68 (0.67;0.68) | 0.68 (0.67;0.68) | 1.49 (1.13;1.95) | 5.42 (4.37;6.73) |
| **25** | Mild liver disease | 0.50 (0.50;0.50) | 0.68 (0.67;0.68) | 0.68 (0.67;0.68) | 1.62 (1.15;2.29) | 2.35 (1.72;3.20) |
| **26** | Number of children = 0 | 0.51 (0.51;0.52) | 0.68 (0.67;0.68) | 0.68 (0.67;0.68) | 0.89 (0.84;0.95) | 0.88 (0.84;0.92) |
| **27** | Muscle pain | 0.52 (0.52;0.53) | 0.68 (0.67;0.68) | 0.68 (0.67;0.68) | 1.17 (1.05;1.31) | 3.35 (3.06;3.67) |
| **28** | Nerve damage | 0.51 (0.51;0.51) | 0.68 (0.67;0.68) | 0.68 (0.67;0.68) | 0.81 (0.69;0.95) | 2.34 (2.05;2.66) |
| **29** | Cardiovascular disease | 0.50 (0.50;0.50) | 0.68 (0.67;0.68) | 0.68 (0.67;0.68) | 1.13 (1.03;1.25) | 1.08 (0.99;1.18) |
| **30** | 3^rd^ quartile income | 0.50 (0.50;0.51) | 0.68 (0.67;0.68) | 0.68 (0.67;0.68) | 0.94 (0.89;0.99) | 0.96 (0.91;1.00) |
| **31** | BMI (kg/m^2^) [25,30] | 0.50 (0.50;0.51) | 0.68 (0.67;0.68) | 0.68 (0.67;0.68) | 1.15 (1.07;1.24) | 1.00 (0.96;1.05) |
| **32** | BMI (kg/m^2^) [30,35] | 0.53 (0.52;0.53) | 0.68 (0.67;0.68) | 0.68 (0.67;0.68) | 1.20 (1.05;1.36) | 1.48 (1.41;1.56) |
| **33** | Non-smoker | 0.51 (0.51;0.51) | 0.68 (0.67;0.69) | 0.68 (0.67;0.68) | 1.09 (1.01;1.17) | 1.32 (1.24;1.42) |
| **34** | Number of children = 2 | 0.50 (0.50;0.50) | 0.68 (0.67;0.69) | 0.68 (0.67;0.68) | 0.93 (0.86;1.00) | 0.99 (0.94;1.05) |
| **35** | Age [80, . ) | 0.50 (0.50;0.50) | 0.68 (0.67;0.69) | 0.68 (0.67;0.68) | - | 0.83 (0.72;0.95) |
| **36** | Sore throat | 0.51 (0.50;0.51) | 0.68 (0.67;0.69) | 0.68 (0.67;0.68) | - | 4.98 (4.02;6.18) |
| **37** | Smoker | 0.51 (0.50;0.51) | 0.68 (0.67;0.69) | 0.68 (0.67;0.68) | - | 1.07 (1.02;1.13) |
| **38** | Migraine | 0.51 (0.51;0.52) | 0.68 (0.67;0.69) | 0.68 (0.67;0.68) | - | 3.41 (3.03;3.84) |
| **39** | Number of children = 1 | 0.51 (0.51;0.52) | 0.68 (0.67;0.69) | 0.68 (0.67;0.68) | - | 1.21 (1.14;1.27) |
| **40** | Chronic obstructive pulmonary disease | 0.50 (0.50;0.51) | 0.68 (0.67;0.69) | 0.68 (0.67;0.68) | - | 1.44 (1.26;1.65) |
| **41** | Depression | 0.52 (0.52;0.52) | 0.68 (0.67;0.69) | 0.68 (0.67;0.68) | - | 1.97 (1.83;2.12) |
| **42** | Type-1 Diabetes Mellitus | 0.50 (0.50;0.50) | 0.68 (0.67;0.69) | 0.68 (0.67;0.68) | - | 0.96 (0.73;1.26) |
| **43** | Arthritis | 0.51 (0.51;0.51) | 0.68 (0.67;0.69) | 0.68 (0.67;0.68) | - | 2.30 (2.06;2.57) |
| **44** | Asplenia | 0.50 (0.50;0.50) | 0.68 (0.67;0.69) | 0.68 (0.67;0.68) | - | 3.71 (1.00;13.82) |
| **45** | Osteoarthritis | 0.52 (0.52;0.52) | 0.68 (0.67;0.69) | 0.68 (0.67;0.68) | - | 2.31 (2.12;2.51) |
| **46** | Shoulder or neck pain | 0.53 (0.52;0.53) | 0.68 (0.67;0.69) | 0.68 (0.67;0.68) | - | 3.02 (2.78;3.28) |
| **47** | Other nerve disease | 0.50 (0.50;0.50) | 0.68 (0.67;0.69) | 0.68 (0.67;0.68) | - | 2.80 (2.20;3.58) |
| **48** | Dementia | 0.50 (0.50;0.50) | 0.68 (0.67;0.69) | 0.68 (0.67;0.68) | - | 1.61 (1.02;2.54) |
| **49** | Postoperative syndrome | 0.50 (0.50;0.50) | 0.68 (0.67;0.69) | 0.68 (0.67;0.68) | - | 3.00 (1.41;6.41) |
| **50** | 2^nd^ quartile income | 0.53 (0.52;0.53) | 0.68 (0.67;0.69) | 0.68 (0.67;0.68) | - | 1.30 (1.24;1.36) |
| **51** | Educational level missing | 0.50 (0.50;0.50) | 0.68 (0.67;0.69) | 0.68 (0.67;0.68) | - | 1.55 (1.24;1.93) |
| **52** | Malicious tumor | 0.50 (0.50;0.50) | 0.68 (0.67;0.69) | 0.68 (0.67;0.68) | - | 1.03 (0.87;1.21) |
| **53** | Hypertension | 0.51 (0.50;0.51) | 0.68 (0.67;0.69) | 0.68 (0.67;0.68) | - | 1.08 (1.03;1.14) |
| **54** | Postoperative pain | 0.50 (0.50;0.51) | 0.68 (0.67;0.69) | 0.68 (0.67;0.68) | - | 2.28 (1.89;2.74) |
| **55** | Other headaches | 0.51 (0.51;0.51) | 0.68 (0.67;0.69) | 0.68 (0.67;0.68) | - | 3.10 (2.74;3.51) |
| **56** | Joint pain | 0.52 (0.52;0.52) | 0.68 (0.67;0.69) | 0.68 (0.67;0.68) | - | 2.79 (2.55;3.05) |
| **57** | Income missing | 0.50 (0.50;0.50) | 0.68 (0.67;0.69) | 0.68 (0.67;0.68) | - | 0.97 (0.70;1.36) |
| **58** | Chronic kidney disease | 0.50 (0.50;0.50) | 0.68 (0.67;0.69) | 0.68 (0.67;0.68) | - | 1.36 (1.04;1.77) |

**Table s4**: Cross-validated forward stepwise prediction model of all predictors for the pre-COVID pain group (n=9,090). The predictors have been ranked by Model 2 and include predictors also ranked after the model cutoff. For each predictor, the values for the univariate and stepwise Area Under the Curve (AUC), the cross-validated stepwise values, the odds ratios from Model 2, and the univariate odds ratios are depicted.

|  | **Predictor** | **Univariate AUC** | **Stepwise**  **AUC** | **Cross-validated stepwise AUC** | **Model 2 odds ratio (CI)** | **Univariate odds**  **ratios (CI)** |
| --- | --- | --- | --- | --- | --- | --- |
| **1** | Use of pain medicine | 0.64 (0.63;0.65) | 0.64 (0.63;0.65) | 0.63 (0.62;0.64) | 3.49 (3.16;3.85) | 3.63 (3.30;3.99) |
| **2** | Breathing pain | 0.52 (0.51;0.52) | 0.65 (0.63;0.66) | 0.64 (0.62;0.65) | 1.56 (1.19;2.05) | 2.49 (1.98;3.13) |
| **3** | Stress | 0.53 (0.52;0.53) | 0.65 (0.64;0.67) | 0.65 (0.63;0.66) | 1.31 (1.13;1.53) | 1.66 (1.46;1.90) |
| **4** | Height | 0.54 (0.53;0.55) | 0.67 (0.65;0.68) | 0.66 (0.65;0.68) | 0.98 (0.98;0.99) | 0.98 (0.98;0.99) |
| **5** | Physical activity | 0.52 (0.51;0.53) | 0.67 (0.66;0.68) | 0.67 (0.66;0.68) | 1.48 (1.31;1.68) | 1.36 (1.21;1.53) |
| **6** | Weight | 0.51 (0.49;0.52) | 0.68 (0.67;0.69) | 0.68 (0.66;0.69) | 1.00 (1.00;1.01) | 1.00 (1.00;1.00) |
| **7** | Age [60,80] | 0.52 (0.51;0.53) | 0.68 (0.67;0.69) | 0.68 (0.67;0.69) | 0.84 (0.76;0.93) | 0.83 (0.76;0.91) |
| **8** | Stomach pain | 0.52 (0.51;0.52) | 0.68 (0.67;0.69) | 0.68 (0.67;0.69) | 1.31 (1.09;1.57) | 1.66 (1.40;1.95) |
| **9** | Type-2 Diabetes Mellitus | 0.51 (0.51;0.52) | 0.68 (0.67;0.69) | 0.68 (0.67;0.69) | 1.34 (1.12;1.60) | 1.41 (1.20;1.66) |
| **10** | Breast pain | 0.52 (0.51;0.52) | 0.68 (0.67;0.69) | 0.68 (0.67;0.69) | 1.44 (1.11;1.87) | 2.35 (1.89;2.93) |
| **11** | 4^th^ quartile income | 0.52 (0.51;0.53) | 0.68 (0.67;0.70) | 0.68 (0.67;0.69) | 0.84 (0.72;0.98) | 0.66 (0.58;0.76) |
| **12** | Whiplash | 0.51 (0.51;0.52) | 0.68 (0.67;0.70) | 0.68 (0.67;0.69) | 1.28 (1.08;1.51) | 1.37 (1.17;1.61) |
| **13** | Mild liver disease | 0.50 (0.50;0.51) | 0.68 (0.67;0.70) | 0.68 (0.67;0.69) | 2.32 (1.26;4.28) | 2.97 (1.68;5.24) |
| **14** | Anxiety | 0.52 (0.52;0.53) | 0.69 (0.67;0.70) | 0.68 (0.67;0.69) | 1.23 (1.04;1.45) | 1.66 (1.44;1.91) |
| **15** | BMI (kg/m^2^) [30,35] | 0.52 (0.51;0.53) | 0.69 (0.67;0.70) | 0.68 (0.67;0.69) | 1.17 (1.04;1.32) | 1.25 (1.12;1.39) |
| **16** | Arthritis | 0.50 (0.50;0.51) | 0.69 (0.68;0.70) | 0.68 (0.67;0.70) | 0.83 (0.73;0.94) | 0.94 (0.84;1.06) |
| **17** | Osteoarthritis | 0.51 (0.50;0.52) | 0.69 (0.68;0.70) | 0.68 (0.67;0.70) | 0.86 (0.78;0.96) | 0.91 (0.83;1.00) |
| **18** | Muscle pain | 0.53 (0.53;0.54) | 0.69 (0.68;0.70) | 0.69 (0.67;0.70) | 1.16 (1.03;1.30) | 1.48 (1.34;1.64) |
| **19** | Nerve damage | 0.50 (0.49;0.51) | 0.69 (0.68;0.70) | 0.69 (0.67;0.70) | 0.85 (0.73;0.98) | 0.97 (0.85;1.11) |
| **20** | Higher education | 0.53 (0.52;0.54) | 0.69 (0.68;0.70) | 0.69 (0.67;0.70) | 0.82 (0.72;0.93) | 0.80 (0.73;0.87) |
| **21** | Medium education | 0.50 (0.49;0.51) | 0.69 (0.68;0.70) | 0.69 (0.67;0.70) | 0.87 (0.77;0.99) | 1.03 (0.94;1.12) |
| **22** | Non-mild liver disease | 0.50 (0.50;0.50) | 0.69 (0.68;0.70) | 0.69 (0.68;0.70) | 2.11 (1.00;4.48) | 2.54 (1.26;5.11) |
| **23** | Smoker | 0.51 (0.51;0.52) | 0.69 (0.68;0.70) | 0.69 (0.68;0.70) | 0.90 (0.81;1.00) | 0.85 (0.77;0.94) |
| **24** | Asthma | 0.52 (0.51;0.52) | 0.69 (0.68;0.70) | 0.69 (0.68;0.70) | 1.15 (0.99;1.33) | 1.39 (1.22;1.59) |
| **25** | Non-smoker | 0.50 (0.49;0.51) | 0.69 (0.68;0.70) | 0.69 (0.68;0.70) | - | 1.04 (0.91;1.18) |
| **26** | Number of children = 1 | 0.51 (0.51;0.52) | 0.69 (0.68;0.70) | 0.69 (0.68;0.70) | - | 1.23 (1.09;1.38) |
| **27** | Type-1 Diabetes Mellitus | 0.50 (0.50;0.50) | 0.69 (0.68;0.71) | 0.69 (0.68;0.70) | - | 0.83 (0.52;1.33) |
| **28** | Non-cohabitant | 0.51 (0.50;0.53) | 0.69 (0.68;0.71) | 0.69 (0.68;0.70) | - | 1.12 (1.03;1.23) |
| **29** | BMI (kg/m^2^) [40, . ) | 0.50 (0.50;0.51) | 0.69 (0.68;0.71) | 0.69 (0.67;0.70) | - | 1.13 (0.91;1.40) |
| **30** | Age [80, . ) | 0.50 (0.50;0.51) | 0.69 (0.68;0.71) | 0.69 (0.67;0.70) | - | 0.79 (0.60;1.04) |
| **31** | Chronic obstructive pulmonary disease | 0.50 (0.50;0.51) | 0.69 (0.68;0.71) | 0.69 (0.67;0.70) | - | 1.29 (1.02;1.63) |
| **32** | Back pain | 0.52 (0.51;0.53) | 0.69 (0.68;0.71) | 0.69 (0.67;0.70) | - | 1.17 (1.07;1.27) |
| **33** | Sore throat | 0.51 (0.51;0.52) | 0.69 (0.68;0.71) | 0.69 (0.67;0.70) | - | 2.15 (1.73;2.68) |
| **34** | Depression | 0.52 (0.51;0.52) | 0.69 (0.68;0.71) | 0.69 (0.67;0.70) | - | 1.34 (1.17;1.53) |
| **35** | 2^nd^ quartile income | 0.51 (0.50;0.52) | 0.69 (0.68;0.71) | 0.69 (0.67;0.70) | - | 1.13 (1.02;1.24) |
| **36** | Female (CPR) | 0.53 (0.52;0.54) | 0.69 (0.68;0.71) | 0.69 (0.67;0.70) | - | 1.32 (1.20;1.46) |
| **37** | Other headaches | 0.52 (0.51;0.52) | 0.70 (0.68;0.71) | 0.69 (0.67;0.70) | - | 1.33 (1.17;1.52) |
| **38** | Hypertension | 0.50 (0.49;0.51) | 0.70 (0.68;0.71) | 0.69 (0.67;0.70) | - | 1.01 (0.91;1.11) |
| **39** | Migraine | 0.52 (0.52;0.53) | 0.70 (0.68;0.71) | 0.69 (0.67;0.70) | - | 1.48 (1.31;1.68) |
| **40** | Age [40,60] | 0.52 (0.51;0.53) | 0.70 (0.68;0.71) | 0.69 (0.67;0.70) | - | 1.21 (1.11;1.32) |
| **41** | Income missing | 0.50 (0.50;0.50) | 0.70 (0.68;0.71) | 0.68 (0.67;0.70) | - | 0.89 (0.40;1.95) |
| **42** | Number of children = 2 | 0.50 (0.50;0.51) | 0.70 (0.68;0.71) | 0.68 (0.67;0.70) | - | 0.96 (0.84;1.08) |
| **43** | Number of children = 0 | 0.52 (0.51;0.53) | 0.70 (0.68;0.71) | 0.68 (0.67;0.70) | - | 0.87 (0.79;0.95) |
| **44** | Shoulder or neck pain | 0.53 (0.52;0.54) | 0.70 (0.68;0.71) | 0.68 (0.67;0.70) | - | 1.30 (1.19;1.44) |
| **45** | Postoperative syndrome | 0.50 (0.50;0.50) | 0.70 (0.68;0.71) | 0.68 (0.67;0.70) | - | 2.96 (0.83;10.48) |
| **46** | Asplenia | 0.50 (0.50;0.50) | 0.70 (0.68;0.71) | 0.68 (0.67;0.70) | - | 1.97 (0.28;13.98) |
| **47** | Postoperative pain | 0.50 (0.50;0.51) | 0.70 (0.68;0.71) | 0.68 (0.67;0.70) | - | 0.96 (0.79;1.15) |
| **48** | BMI (kg/m^2^) [35,40] | 0.51 (0.50;0.51) | 0.70 (0.68;0.71) | 0.68 (0.67;0.70) | - | 1.25 (1.07;1.47) |
| **49** | Educational level missing | 0.50 (0.50;0.50) | 0.70 (0.68;0.71) | 0.68 (0.67;0.70) | - | 1.46 (0.98;2.17) |
| **50** | Malicious tumor | 0.50 (0.50;0.50) | 0.70 (0.68;0.71) | 0.68 (0.67;0.70) | - | 0.89 (0.65;1.22) |
| **51** | BMI (kg/m^2^) [25,30] | 0.51 (0.50;0.52) | 0.70 (0.68;0.71) | 0.68 (0.67;0.70) | - | 0.93 (0.85;1.02) |
| **52** | Joint pain | 0.52 (0.51;0.52) | 0.70 (0.68;0.71) | 0.68 (0.67;0.69) | - | 1.18 (1.07;1.30) |
| **53** | 3^rd^ quartile income | 0.51 (0.50;0.52) | 0.70 (0.68;0.71) | 0.68 (0.67;0.69) | - | 0.86 (0.77;0.96) |
| **54** | Other nerve disease | 0.50 (0.50;0.51) | 0.70 (0.68;0.71) | 0.68 (0.67;0.69) | - | 1.19 (0.93;1.52) |
| **55** | Chronic kidney disease | 0.50 (0.50;0.50) | 0.70 (0.68;0.71) | 0.68 (0.67;0.69) | - | 1.39 (0.88;2.21) |
| **56** | Dementia | 0.50 (0.50;0.50) | 0.70 (0.68;0.71) | 0.68 (0.67;0.69) | - | 1.22 (0.61;2.44) |
| **57** | Neurological symptoms | 0.50 (0.50;0.51) | 0.70 (0.68;0.71) | 0.68 (0.67;0.69) | - | 1.10 (0.95;1.26) |
| **58** | Cardiovascular disease | 0.50 (0.50;0.51) | 0.70 (0.68;0.71) | 0.68 (0.67;0.69) | - | 1.01 (0.85;1.21) |

**Table s5**: Cross-validated forward stepwise prediction model of all predictors for the non-pre-COVID pain group (n=55,938). The pain variables are excluded from this subgroup as they reported no pre-COVID pain. The predictors have been ranked by Model 3 and include predictors also ranked after the model cutoff. For each predictor, the values for the univariate and stepwise Area Under the Curve (AUC), the cross-validated stepwise values, the odds ratios from Model 3, and the univariate odds ratios are depicted.

|  | **Predictor** | **Univariate AUC** | **Stepwise**  **AUC** | **Cross-validated stepwise AUC** | **Model 3 odds ratio (CI)** | **Univariate odds**  **ratios (CI)** |
| --- | --- | --- | --- | --- | --- | --- |
| **1** | Stress | 0.53 (0.52;0.53) | 0.53 (0.52;0.53) | 0.52 (0.51;0.53) | 1.83 (1.66;2.02) | 2.43 (2.23;2.65) |
| **2** | Female (CPR) | 0.55 (0.55;0.56) | 0.57 (0.57;0.58) | 0.57 (0.56;0.57) | 1.49 (1.39;1.60) | 1.53 (1.46;1.61) |
| **3** | Weight | 0.54 (0.53;0.55) | 0.61 (0.60;0.62) | 0.61 (0.60;0.62) | 1.02 (1.01;1.02) | 1.01 (1.01;1.01) |
| **4** | Higher education | 0.54 (0.54;0.55) | 0.62 (0.61;0.63) | 0.62 (0.61;0.62) | 0.66 (0.61;0.71) | 0.71 (0.68;0.74) |
| **5** | Age [40,60] | 0.54 (0.53;0.55) | 0.63 (0.62;0.63) | 0.62 (0.62;0.63) | 1.27 (1.18;1.36) | 1.38 (1.31;1.44) |
| **6** | 4^th^ quartile income | 0.55 (0.54;0.55) | 0.63 (0.63;0.64) | 0.63 (0.62;0.64) | 0.75 (0.70;0.80) | 0.60 (0.57;0.64) |
| **7** | Physical activity | 0.52 (0.51;0.52) | 0.64 (0.63;0.64) | 0.64 (0.63;0.64) | 1.59 (1.48;1.71) | 1.31 (1.22;1.40) |
| **8** | Height | 0.55 (0.54;0.56) | 0.64 (0.63;0.65) | 0.64 (0.63;0.65) | 0.98 (0.98;0.99) | 0.98 (0.98;0.98) |
| **9** | Asthma | 0.52 (0.51;0.52) | 0.64 (0.64;0.65) | 0.64 (0.63;0.65) | 1.41 (1.30;1.53) | 1.55 (1.43;1.68) |
| **10** | Neurological symptoms | 0.51 (0.50;0.51) | 0.64 (0.64;0.65) | 0.64 (0.64;0.65) | 1.79 (1.52;2.12) | 2.00 (1.70;2.34) |
| **11** | Anxiety | 0.52 (0.51;0.52) | 0.64 (0.64;0.65) | 0.64 (0.64;0.65) | 1.26 (1.12;1.41) | 2.05 (1.86;2.27) |
| **12** | Medium education | 0.52 (0.52;0.53) | 0.64 (0.64;0.65) | 0.64 (0.64;0.65) | 0.83 (0.77;0.89) | 1.21 (1.16;1.27) |
| **13** | Age [60,80] | 0.54 (0.53;0.54) | 0.65 (0.64;0.65) | 0.64 (0.64;0.65) | 0.81 (0.75;0.88) | 0.72 (0.69;0.76) |
| **14** | BMI (kg/m^2^) [40, . ) | 0.51 (0.51;0.51) | 0.65 (0.64;0.65) | 0.64 (0.64;0.65) | 0.79 (0.65;0.96) | 2.06 (1.79;2.37) |
| **15** | Non-smoker | 0.51 (0.51;0.51) | 0.65 (0.64;0.65) | 0.65 (0.64;0.65) | 1.18 (1.08;1.29) | 1.30 (1.20;1.41) |
| **16** | BMI (kg/m^2^) [25,30] | 0.50 (0.50;0.51) | 0.65 (0.64;0.65) | 0.65 (0.64;0.65) | 1.14 (1.08;1.21) | 1.04 (0.99;1.09) |
| **17** | Non-cohabitant | 0.52 (0.51;0.53) | 0.65 (0.64;0.65) | 0.65 (0.64;0.65) | 1.09 (1.04;1.15) | 1.19 (1.13;1.24) |
| **18** | Non-mild liver disease | 0.50 (0.50;0.50) | 0.65 (0.64;0.65) | 0.65 (0.64;0.65) | 2.60 (1.41;4.80) | 3.26 (1.82;5.83) |
| **19** | Cardiovascular disease | 0.50 (0.50;0.50) | 0.65 (0.64;0.65) | 0.65 (0.64;0.65) | 1.17 (1.05;1.31) | 1.05 (0.94;1.16) |
| **20** | BMI (kg/m^2^) [30,35] | 0.52 (0.52;0.53) | 0.65 (0.64;0.65) | 0.65 (0.64;0.65) | 1.13 (1.04;1.23) | 1.45 (1.37;1.54) |
| **21** | Depression | 0.52 (0.51;0.52) | 0.65 (0.64;0.66) | 0.65 (0.64;0.65) | 1.17 (1.05;1.30) | 1.89 (1.73;2.08) |
| **22** | Type-2 Diabetes Mellitus | 0.51 (0.50;0.51) | 0.65 (0.64;0.66) | 0.65 (0.64;0.65) | 1.15 (1.03;1.29) | 1.26 (1.14;1.40) |
| **23** | Smoker | 0.51 (0.50;0.51) | 0.65 (0.64;0.66) | 0.65 (0.64;0.65) | 1.08 (1.02;1.14) | 1.10 (1.04;1.16) |
| **24** | Number of children = 0 | 0.51 (0.51;0.52) | 0.65 (0.64;0.66) | 0.65 (0.64;0.65) | - | 0.88 (0.84;0.92) |
| **25** | 3^rd^ quartile income | 0.50 (0.50;0.51) | 0.65 (0.64;0.66) | 0.65 (0.64;0.65) | - | 1.04 (0.99;1.10) |
| **26** | Age [80, . ) | 0.50 (0.50;0.50) | 0.65 (0.64;0.66) | 0.65 (0.64;0.65) | - | 0.82 (0.70;0.96) |
| **27** | Number of children = 2 | 0.50 (0.50;0.51) | 0.65 (0.64;0.66) | 0.65 (0.64;0.65) | - | 1.02 (0.96;1.09) |
| **28** | Number of children = 1 | 0.51 (0.51;0.51) | 0.65 (0.64;0.66) | 0.65 (0.64;0.65) | - | 1.18 (1.11;1.26) |
| **29** | Mild liver disease | 0.50 (0.50;0.50) | 0.65 (0.64;0.66) | 0.65 (0.64;0.65) | - | 1.70 (1.13;2.56) |
| **30** | Asplenia | 0.50 (0.50;0.50) | 0.65 (0.64;0.66) | 0.65 (0.64;0.65) | - | 3.74 (0.62;22.36) |
| **31** | Chronic obstructive pulmonary disease | 0.50 (0.50;0.50) | 0.65 (0.64;0.66) | 0.65 (0.64;0.65) | - | 1.25 (1.05;1.48) |
| **32** | BMI (kg/m^2^) [35,40] | 0.51 (0.51;0.52) | 0.65 (0.64;0.66) | 0.65 (0.64;0.65) | - | 1.75 (1.59;1.93) |
| **33** | Type-1 Diabetes Mellitus | 0.50 (0.50;0.50) | 0.65 (0.64;0.66) | 0.65 (0.64;0.65) | - | 0.86 (0.61;1.22) |
| **34** | Dementia | 0.50 (0.50;0.50) | 0.65 (0.64;0.66) | 0.65 (0.64;0.65) | - | 1.32 (0.70;2.47) |
| **35** | Malicious tumor | 0.50 (0.50;0.50) | 0.65 (0.64;0.66) | 0.65 (0.64;0.65) | - | 0.99 (0.82;1.21) |
| **36** | Educational level missing | 0.50 (0.50;0.50) | 0.65 (0.64;0.66) | 0.65 (0.64;0.65) | - | 1.38 (1.05;1.81) |
| **37** | Chronic kidney disease | 0.50 (0.50;0.50) | 0.65 (0.64;0.66) | 0.65 (0.64;0.65) | - | 1.14 (0.81;1.59) |
| **38** | 2^nd^ quartile income | 0.52 (0.52;0.53) | 0.65 (0.64;0.66) | 0.65 (0.64;0.65) | - | 1.29 (1.23;1.36) |
| **39** | Hypertension | 0.50 (0.50;0.51) | 0.65 (0.64;0.66) | 0.65 (0.64;0.65) | - | 1.04 (0.98;1.10) |
| **40** | Postoperative syndrome | 0.50 (0.50;0.50) | 0.65 (0.64;0.66) | 0.65 (0.64;0.65) | - | 2.16 (0.77;6.05) |
| **41** | Income missing | 0.50 (0.50;0.50) | 0.65 (0.64;0.66) | 0.65 (0.64;0.65) | - | 1.03 (0.71;1.49) |

**Table s6**: Sensitivity analysis. Cross-validated forward stepwise prediction model of 58 predictors for the full study cohort (n=65,028) using AUCs as selection criteria. The forward selection terminated at 32 predictors. The predictors have been ranked by the model and include predictors also ranked after the stopping point. For each predictor, the cross-validated Area Under the Curve (cvAUC) is predicted.

|  | **Predictor** | **Cross-validated stepwise AUC** | **Stopping point** |
| --- | --- | --- | --- |
| **1** | Height | 0.56 (0.55;0.57) | Included |
| **2** | Weight | 0.60 (0.59;0.61) | Included |
| **3** | Use of pain medicine | 0.63 (0.62;0.64) | Included |
| **4** | Stress | 0.64 (0.64;0.65) | Included |
| **5** | 4^th^ quartile income | 0.65 (0.64;0.66) | Included |
| **6** | Age [40,60] | 0.66 (0.65;0.66) | Included |
| **7** | Physical activity | 0.66 (0.66;0.67) | Included |
| **8** | Back pain | 0.67 (0.66;0.67) | Included |
| **9** | Female (CPR) | 0.67 (0.66;0.67) | Included |
| **10** | Higher education | 0.67 (0.66;0.68) | Included |
| **11** | Asthma | 0.67 (0.67;0.68) | Included |
| **12** | Anxiety | 0.67 (0.67;0.68) | Included |
| **13** | Age [60,80] | 0.67 (0.67;0.68) | Included |
| **14** | Neurological symptoms | 0.67 (0.67;0.68) | Included |
| **15** | Muscle pain | 0.67 (0.67;0.68) | Included |
| **16** | Type-2 Diabetes Mellitus | 0.68 (0.67;0.68) | Included |
| **17** | Breathing pain | 0.68 (0.67;0.68) | Included |
| **18** | BMI (kg/m^2^) [40, . ) | 0.68 (0.67;0.68) | Included |
| **19** | Medium education | 0.68 (0.67;0.68) | Included |
| **20** | Non-mild liver disease | 0.68 (0.67;0.68) | Included |
| **21** | Non-cohabitant | 0.68 (0.67;0.68) | Included |
| **22** | Whiplash | 0.68 (0.67;0.68) | Included |
| **23** | BMI (kg/m^2^) [25,30] | 0.68 (0.67;0.68) | Included |
| **24** | Non-smoker | 0.68 (0.67;0.68) | Included |
| **25** | Stomach pain | 0.68 (0.67;0.68) | Included |
| **26** | Depression | 0.68 (0.67;0.68) | Included |
| **27** | Smoker | 0.68 (0.67;0.68) | Included |
| **28** | Mild liver disease | 0.68 (0.67;0.68) | Included |
| **29** | Migraine | 0.68 (0.67;0.68) | Included |
| **30** | 2^nd^ quartile income | 0.68 (0.67;0.68) | Included |
| **31** | BMI (kg/m^2^) [30,35] | 0.68 (0.67;0.68) | Included |
| **32** | Nerve damage | 0.68 (0.67;0.68) | Stopping point (included) |
| **33** | Age [80, . ) | 0.68 (0.67;0.68) | - |
| **34** | Chronic obstructive pulmonary disease | 0.68 (0.67;0.68) | - |
| **35** | Sore throat | 0.68 (0.67;0.68) | - |
| **36** | Educational level missing | 0.68 (0.67;0.68) | - |
| **37** | Number of children = 1 | 0.68 (0.67;0.68) | - |
| **38** | Shoulder or neck pain | 0.68 (0.67;0.68) | - |
| **39** | Cardiovascular disease | 0.68 (0.67;0.68) | - |
| **40** | Hypertension | 0.68 (0.67;0.68) | - |
| **41** | 3^rd^ quartile income | 0.68 (0.67;0.68) | - |
| **42** | Dementia | 0.68 (0.67;0.68) | - |
| **43** | Postoperative syndrome | 0.68 (0.67;0.68) | - |
| **44** | Malicious tumor | 0.68 (0.67;0.68) | - |
| **45** | Other nerve disease | 0.68 (0.67;0.68) | - |
| **46** | BMI (kg/m^2^) [35,40] | 0.68 (0.67;0.68) | - |
| **47** | Number of children = 2 | 0.68 (0.67;0.68) | - |
| **48** | Number of children = 0 | 0.68 (0.67;0.68) | - |
| **49** | Chronic kidney disease | 0.68 (0.67;0.68) | - |
| **50** | Joint pain | 0.68 (0.67;0.68) | - |
| **51** | Asplenia | 0.68 (0.67;0.68) | - |
| **52** | Postoperative pain | 0.68 (0.67;0.68) | - |
| **53** | Income missing | 0.68 (0.67;0.68) | - |
| **54** | Arthritis | 0.68 (0.67;0.68) | - |
| **55** | Other headaches | 0.68 (0.67;0.68) | - |
| **56** | Breast pain | 0.68 (0.67;0.68) | - |
| **57** | Osteoarthritis | 0.68 (0.67;0.68) | - |
| **58** | Type-1 Diabetes Mellitus | 0.68 (0.67;0.68) | - |

**Table s7**: Sensitivity analysis. Cross-validated forward stepwise prediction model of 58 predictors for the pre-COVID pain group (n=9,090) using AUCs as selection criteria. The forward selection terminated at 25 predictors. The predictors have been ranked by the model and include predictors also ranked after the stopping point. For each predictor, the cross-validated Area Under the Curve (cvAUC) is predicted.

|  | **Predictor** | **Cross-validated stepwise AUC** | **Stopping point** |
| --- | --- | --- | --- |
| **1** | Use of pain medicine | 0.63 (0.62;0.64) | Included |
| **2** | Height | 0.65 (0.64;0.67) | Included |
| **3** | Stress | 0.66 (0.65;0.67) | Included |
| **4** | Physical activity | 0.67 (0.65;0.68) | Included |
| **5** | Weight | 0.67 (0.66;0.68) | Included |
| **6** | Breathing pain | 0.68 (0.66;0.69) | Included |
| **7** | Age [60,80] | 0.68 (0.67;0.69) | Included |
| **8** | Type-2 Diabetes Mellitus | 0.68 (0.67;0.69) | Included |
| **9** | Osteoarthritis | 0.68 (0.67;0.69) | Included |
| **10** | 4^th^ quartile income | 0.68 (0.67;0.69) | Included |
| **11** | Anxiety | 0.68 (0.67;0.69) | Included |
| **12** | Mild liver disease | 0.68 (0.67;0.69) | Included |
| **13** | Arthritis | 0.68 (0.67;0.70) | Included |
| **14** | Sore throat | 0.68 (0.67;0.70) | Included |
| **15** | Higher education | 0.69 (0.67;0.70) | Included |
| **16** | Muscle pain | 0.69 (0.67;0.70) | Included |
| **17** | Stomach pain | 0.69 (0.67;0.70) | Included |
| **18** | Non-mild liver disease | 0.69 (0.67;0.70) | Included |
| **19** | BMI (kg/m^2^) [30,35] | 0.69 (0.68;0.70) | Included |
| **20** | Asthma | 0.69 (0.68;0.70) | Included |
| **21** | Nerve damage | 0.69 (0.68;0.70) | Included |
| **22** | Medium education | 0.69 (0.68;0.70) | Included |
| **23** | Smoker | 0.69 (0.68;0.70) | Included |
| **24** | Back pain | 0.69 (0.68;0.70) | Included |
| **25** | 2^nd^ quartile income | 0.69 (0.68;0.70) | Stopping point (included) |
| **26** | Depression | 0.69 (0.68;0.70) | - |
| **27** | Hypertension | 0.69 (0.68;0.70) | - |
| **28** | Number of children = 1 | 0.69 (0.68;0.70) | - |
| **29** | Non-smoker | 0.69 (0.68;0.70) | - |
| **30** | Type-1 Diabetes Mellitus | 0.69 (0.68;0.70) | - |
| **31** | Malicious tumor | 0.69 (0.68;0.70) | - |
| **32** | BMI (kg/m^2^) [40, . ) | 0.69 (0.68;0.70) | - |
| **33** | Female (CPR) | 0.69 (0.68;0.70) | - |
| **34** | Number of children = 2 | 0.69 (0.68;0.70) | - |
| **35** | Age [40,60] | 0.69 (0.68;0.70) | - |
| **36** | Other headaches | 0.69 (0.68;0.70) | - |
| **37** | Educational level missing | 0.69 (0.68;0.70) | - |
| **38** | Asplenia | 0.69 (0.68;0.70) | - |
| **39** | Breast pain | 0.69 (0.68;0.70) | - |
| **40** | Non-cohabitant | 0.69 (0.68;0.70) | - |
| **41** | Postoperative pain | 0.69 (0.68;0.70) | - |
| **42** | Income missing | 0.69 (0.68;0.70) | - |
| **43** | Dementia | 0.69 (0.68;0.70) | - |
| **44** | Chronic kidney disease | 0.69 (0.68;0.70) | - |
| **45** | BMI (kg/m^2^) [25,30] | 0.69 (0.68;0.70) | - |
| **46** | Neurological symptoms | 0.69 (0.67;0.70) | - |
| **47** | Cardiovascular disease | 0.69 (0.67;0.70) | - |
| **48** | 3^rd^ quartile income | 0.69 (0.67;0.70) | - |
| **49** | Joint pain | 0.69 (0.67;0.70) | - |
| **50** | Postoperative syndrome | 0.69 (0.67;0.70) | - |
| **51** | Other nerve disease | 0.69 (0.67;0.70) | - |
| **52** | Chronic obstructive pulmonary disease | 0.69 (0.67;0.70) | - |
| **53** | Whiplash | 0.69 (0.67;0.70) | - |
| **54** | Migraine | 0.69 (0.67;0.70) | - |
| **55** | Shoulder or neck pain | 0.69 (0.67;0.70) | - |
| **56** | BMI (kg/m^2^) [35,40] | 0.69 (0.67;0.70) | - |
| **57** | Age [80, . ) | 0.69 (0.67;0.70) | - |
| **58** | Number of children = 0 | 0.68 (0.67;0.70) | - |

**Table s8**: Sensitivity analysis. Cross-validated forward stepwise prediction model of 58 predictors for the non-pre-COVID pain group (n=55,938) using AUCs as selection criteria. The forward selection terminated at 27 predictors. The predictors have been ranked by the model and include predictors also ranked after the model cutoff. For each predictor, the cross-validated Area Under the Curve (cvAUC) is predicted.

|  | **Predictor** | **Cross-validated stepwise AUC** | **Stopping point** |
| --- | --- | --- | --- |
| **1** | Female (CPR) | 0.55 (0.54;0.56) | Included |
| **2** | Weight | 0.59 (0.59;0.60) | Included |
| **3** | Stress | 0.61 (0.60;0.62) | Included |
| **4** | Higher education | 0.62 (0.61;0.62) | Included |
| **5** | Age [40,60) | 0.62 (0.62;0.63) | Included |
| **6** | 4th quartile income | 0.63 (0.63;0.64) | Included |
| **7** | Physical activity | 0.64 (0.63;0.64) | Included |
| **8** | Height | 0.64 (0.63;0.65) | Included |
| **9** | Asthma | 0.64 (0.63;0.65) | Included |
| **10** | Age [60,80) | 0.64 (0.64;0.65) | Included |
| **11** | Neurological symptoms | 0.64 (0.64;0.65) | Included |
| **12** | Anxiety | 0.64 (0.64;0.65) | Included |
| **13** | BMI [25,30) | 0.64 (0.64;0.65) | Included |
| **14** | Non-smoker | 0.65 (0.64;0.65) | Included |
| **15** | Depression | 0.65 (0.64;0.65) | Included |
| **16** | Type-2 Diabetes Mellitus | 0.65 (0.64;0.65) | Included |
| **17** | Medium education | 0.65 (0.64;0.65) | Included |
| **18** | BMI [30,35) | 0.65 (0.64;0.65) | Included |
| **19** | Non-cohabitant | 0.65 (0.64;0.65) | Included |
| **20** | Smoker | 0.65 (0.64;0.65) | Included |
| **21** | Non-mild liver disease | 0.65 (0.64;0.65) | Included |
| **22** | Cardiovascular disease | 0.65 (0.64;0.65) | Included |
| **23** | BMI [40, . ) | 0.65 (0.64;0.65) | Included |
| **24** | Age [80, . ) | 0.65 (0.64;0.65) | Included |
| **25** | 3rd quartile income | 0.65 (0.64;0.65) | Included |
| **26** | Mild liver disease | 0.65 (0.64;0.65) | Included |
| **27** | Chronic obstructive pulmonary disease | 0.65 (0.64;0.65) | Stopping point (included) |
| **28** | Educational level missing | 0.65 (0.64;0.65) | - |
| **29** | Dementia | 0.65 (0.64;0.65) | - |
| **30** | Number of children = 1 | 0.65 (0.64;0.65) | - |
| **31** | Type-1 Diabetes Mellitus | 0.65 (0.64;0.65) | - |
| **32** | Malicious tumor | 0.65 (0.64;0.65) | - |
| **33** | Hypertension | 0.65 (0.64;0.65) | - |
| **34** | Income missing | 0.65 (0.64;0.65) | - |
| **35** | BMI [35,40) | 0.65 (0.64;0.65) | - |
| **36** | 2nd quartile income | 0.65 (0.64;0.65) | - |
| **37** | Number of children = 2 | 0.65 (0.64;0.65) | - |
| **38** | Number of children = 0 | 0.65 (0.64;0.65) | - |
| **39** | Chronic kidney disease | 0.65 (0.64;0.65) | - |
| **40** | Postoperative syndrome | 0.65 (0.64;0.65) | - |
| **41** | Asplenia | 0.65 (0.64;0.65) | - |
