# Supplementary material for: Predictive Ability of Previous Pain and Disease Conditions on the Presentation of Post‐COVID Pain in a Danish Cohort of Adult COVID‐19 Survivors
Source: Eur J Pain. 2025 Apr 5;29(5):e70021. doi: 10.1002/ejp.70021 (PMC11971649; doi:10.1002/ejp.70021)
Supplement: Supplementary file 8 — Data S1. [file EJP-29-0-s001.pdf]

Full study cohort

dendrogram_text	original_order	median	count1	count2	count3	count4	count5	count6	count7	count8	count9	count10	count11	count12	count13	count14	count15
**Predictor**	**Original order**	**Median**	**1**	**2**	**3**	**4**	**5**	**6**	**7**	**8**	**9**	**10**	**11**	**12**	**13**	**14**	**15**
**Use of pain medicine**	1	1	200														
**Stress**	2	2		195	4	*											
**4th quartile income**	3	3		4	*		4	4	6	3	18	14	5				
**Age [40,60)**	4	4				*		13	19	4	*				*	10	5
**Female (CPR)**	5	5		*	*	19	30	59	3		16	*	5	3	*		
**Weight**	6	6			*	56	23	55	*	*							
**Higher education**	7	5			*		*	18	29	35	8	5	*	*			
**Physical activity**	8	8						13	50	107	27	3					
**Height**	9	9				*	30	*	13	29	68	33	*	*			
**Asthma**	10	11					*	3	3	*	29	44	70	19	*	7	*
**Breathing pain**	11	13.5						*		*	8	24	27	25	14	9	5
**Backpain**	12	17					*			*	9	18	*	18	*	8	13
**Anxiety**	13	14								*	*	9	14	35	*	*	10
**Medium education**	14	15									3	*	4	19	*	37	25
**Age [60,80)**	15	15						*	15	4	*		4	10	15	24	18
**BMI [40, .)**	16	16									*	*	*	10	*	25	36
**Neurological symptoms**	17	16										5	6	*	23	15	23
**T2D**	18	20												*		7	8
**Stomach pain**	19	21									3	5	6	9	4	6	9
**Non-mild livear disease**	20	22.5													3	6	9
**Whiplash**	21	23												*	4	*	4
**Lives alone**	22	22											*	4	5	7	7
**BMI [35,40)**	23	33						*									
**Breast pain**	24	27									4	*	*	4	13	*	*
**Mild liver disease**	25	27													*		4
**Number of kids = 0**	26	30														*	*
**Musclepain**	27	27									*	9	13	13	6	4	
**Nervedamage**	28	30															
**CVD**	29	28															
**3rd quartile income**	30	32													*	4	*
**BMI [25,30)**	31	25								*						*	5
**BMI [30,35)**	32	30				*	10									*	5
**Non-smoker**	33	30															*
**Number of kids = 2**	34	39															
**Age [80, .)**	35	32															
**Sore throat**	36	33										3	3	*	5		4
**Smoker**	37	39															
**Migraine**	38	39												*			
**Number of kids = 1**	39	42															
**COL**	40	39															
**Depression**	41	40														*	*
**T1D**	42	41															
**Arthritis**	43	44.5															
**Asplenia**	44	40.5															
**OA**	45	45															
**Shoulder/neck**	46	47										3	4	*	4	*	*
**Other nervedisease**	47	47															
**Dementia**	48	49															
**Post operative syndrome**	49	48															
**2nd quartile income**	50	49															
**Educational level missing**	51	49															
**Malicious tumor**	52	48															
**Hypertension**	53	50															
**Postoperative pain**	54	50															
**Other headache**	55	48															
**Jointpain**	56	48.5											*				
**Income missing**	57	49															
**CKD**	58	50															

count16	count17	count18	count19	count20	count21	count22	count23	count24	count25	count26	count27	count28	count29	count30	count31	count32	count33	count34	count35	count36
16	17	18	19	20	21	22	23	24	25	26	27	28	29	30	31	32	33	34	35	36
6	5	3	*	*	*	*	*							*						
													*							
												*								
6	3	5	5	8	5	6	*	6	3	5	4		3	*	*	4	*	*		
6	5	10	5	*	5	4	8	6	4	7	4	7	8	5		5	4	*	*	*
23	16	13	10	4	4	*	4	4	*		*				*	*	*			
16	16	*	8	9	3	6	*	*	*	*	*					*	*			
*	18	10	6	4	4	*	4	*	*		*	*	4	*	*			*	*	*
30	*	15	*		4	*	*		*	*		*				*		*	*	*
*	20	14	10	5	14	9	5	3	*	*	*	*	*							
16	20	25	*	19	*	18	14	4	9	7	4	3	*	4		*			3	*
10	*	13	*	*	10	6	8	*	8	*	4	3	5	7	5	9	3	5	4	*
5	7	*	19	17	*	*	13	14	8	8	4	9	6	6	6	3	4	*	3	*
4	16	16	10	15	13	14	*	6	13	14	6	6	3	5	5	3	3	5	6	
4	*	8	9	*	16	14	*	13	6	10	9	5	5	3	3	4	6	5	*	4
	3	5	8	8	10	*	*	9	4	4	*	8	*	*	4	6	4	3	4	8
3	*	8	5	6	7	3	8	*	5	5	6	5	5	5	4	6	4	5	7	3
*	3	6	8	3	9	9	10	18	8	13	14	10	10	8	4	7	5	7	*	*
*	5	*	5	4	6	6	10	8	*	8	9	7	*	7	5	5	8	5	14	7
4	*	3	6	6	5	3	7	3	6	6	5	5	8	5	9	8	3	*	*	3
			*	3	5	8	*	*	9	6	10	16	14	*	17	7	9	*	10	3
*	3	6	5	8	5	7	6	10	*	13	16	14	14	8	8	3	*	4	5	5
*	4	*	*	7	*	5	5	5	8	8	8	7	14	3	7	7	3	5	3	*
6	7	5	9	9	15	*	7	*	13	*	7	5	6	8	8	5	7	5	5	4
*	*	*	*	6	4	5	3	6	*	9	*	14	4	*	6	7	8	10	3	7
*	4	3	4	3	9	*	7	13	4	9	3	8	13	*	4	10	8	9	6	5
					*		*	5	*	4	3	5	7	*	8	7	4	6	6	*
*	*	*	3	3	4	9	6	8	5	*	*	*	4	8	9	9	10	*	7	9
5	3	*	3	10	4	3	*	6	4	6	8	6	5	*	7	5	9	7	4	5
*					*	*	*		3	5	7	5	3	8	6	5	9	7	9	10
			3	*	5	5	4	6	6	4	*	4	8		10	*	3	5	5	6
			*	3	*	*	*	3	5	*	*	3	5	3	5	4	5	4	5	14
			*	*	*	3	*	5	6	*	6	*	5	8	5	8	9	5	5	4
	*			*			*	*	8	3	4	7	3	4	6	3	8	5	5	8
				*			*	3	*	*	3	3	4	7	6	6	3	5	5	10
							*			*	3	*		3	6	8	5	*	5	8
										*	*	*	*	3	7	4	10	7	4	7
						*		*	*	*	3	*	3	4		4	4	6	5	6
*	*	*	*			*	*	*	*					4	*	3	*	3	*	3
									*	*	*	*	*	3	*	5	*	3	7	3
									*	*	*			*	*	*		3	5	3	*
							*			*	*		3	*	*	3	*	*	8	4	7
			*		*	*	3	*	*	*		*	*	3	*	*	*	4	6	5
						*	*	*			5	*	*	3	*	3	4	4	3	4
								*	*	*	*	*			*	4	3	3	3	4
									*	*	*		*	3	3	3	4	*	3	*
										*	*		*	*	*	*	*	3	*	*
												*	*	*		3			*	4
					*					*	*	*	*	*	3	*	4	*	*	3
												*			*	5		3	3	3
		*									*	*	*		*			*	4	*

count37	count38	count39	count40	count41	count42	count43	count44	count45	count46	count47	count48	count49	count50	count51	count52	count53	count54	count55	count56	count57	count58
37	38	39	40	41	42	43	44	45	46	47	48	49	50	51	52	53	54	55	56	57	58
*	*	*	*	*		*		*		*			*								
*	3		*			*	*								*						
				*																	
*				*																	
*			*	*	*						*	*	4			*	*	*	*	*	*
	*																				
*	*	*		*	*	3	*	*	*		3			*			*				
*	*	4		*	*		*		*		*				*						
*	*	*	*	*		*	*	*	*	*			*								
3			*		*	*															
	4	3	3	*	4	3	5	6	3	8	4	*	6	3	3	6	5	*	*	4	5
4	6	5	*	*	4	3	5	3	3	*	*	3	*	*	*	4					
3	*	3	*	*	*	*	*			*	*		*	*	*		*	*			*
4	4	7	5	6	7	5	*	*	*	3	*	*	*	*	*					*	
*	3	*	*	3	5	*	3	*	*	4	3	6	*	*	3	*	*	*	*	4	*
7	*	3	4	4		*	4		*		4	*				*	*		*		
9	4	4	4		*	*	*	*	*	3		*				*					*
4	*	*	3	3	3	5		3	4	4	3	4	6	6	5	5	3	5	3	7	7
4	3	4	*	3	*	*	*	*	*	3	*	*									
6	7	3	6	3	7	*	*	4	*	*	3		*				*			*	
8	6	3	3	9	*	*	6	*	3	*	4			*		*		*	*		
4	9	10	10	8	9	13	9	7	10	5	8	*	4	5		*		*	*	*	*
8	3	4	7	4	4	*	3	8	*	*	3	*	*	*	*	*	*	*	*		
3	6	4	7	4	6	4	9	4	3	3	3	*		*	4	4	*	4		3	3
7	6	9	4	7	8	10	5	8	7	4	6	7	*	3	3	3	4	7	5	*	*
10	7	6	7	5	*	6	*	6	3	5	8	*	7	5	5	5	6	*	8	*	5
4	5	9	6	6	7	15	14	9	6	*	4	9	7	*	*	3	3	3	*	3	
8	10	7	10	5	7	6	7	4	3	*	4	6	7	9	3	*	5	5	5	3	5
8	8	9	6	10	4	8	5	7	*	8	7	4	4	7	4	4	6		4	4	9
*	8	7	8	10	9	4	3	7	8	8	4	6	4	4	*	9	4	7	6	8	3
5	*	5	6	6	8	6	8	8	10	7	9	*	3	8	6	7	6	5	7	7	6
*	*	16	*	4	5	4	3	4	7	7	8	6	6	6	5	5	3	4	8	4	8
5	4	9	10	8	5	6	9	4	6	7	8	6	6	7	7	9	5	9	8	10	9
*	5	4	3	4	5	5	8	7	8	7	8	*	7	10	7	*	10	5	6	9	10
5	3	10	6	7	4	9	8	8	6	7	7	*	5	8	9	5	*	*	*	13	*
5	8	4	8	4	5	5	7	10	9	7	*	14	5	*	9	*	13	*	15	9	5
6	5	5	10	4	6	8	*	6	9	6	14	6	*	3	*	8	6	*	5	*	3
*	6	3	3	8	7	4	6	10	*	5	5	5	7	8	4	13	16	17	9	*	10
3	*	3	8	4	3	6	6	5	14	10	*	10	7	8	10	19	5	17	7	*	7
*	9	*	5	8	*	3	8	10	7	6	7	8	*	8	18	9	*	*	10	8	*
4	5	5	5	8	4	3	7	8	7	5	3	9	*	9	*	4	*	10	17	18	10
3	3	4	3	4	6	8	10	3	4	10	7	8	18	16	10	*	*	8	9	13	14
7	*	7	5	9	5	7	6	7	14	16	6	10	9	15	*	*	9	4	10	6	10
3	4	4	5	4	9	6	7	7	9	7	*	7	10	8	8	*	8	9	14	9	16
6	5	5	3	9	7	8	7	9	9	4	10	10	*	8	17	6	*	9	*	8	8
3	*	3	*	6	5	*	7	5	9	6	14	*	8	13	14	5	*	13	*	7	19

Pre-COVID pain group

dendrogram_text	original_order	median	count1	count2	count3	count4	count5	count6	count7	count8	count9	count10	count11	count12	count13	count14	count15
**Predictor**	**Original order**	**Median**	**1**	**2**	**3**	**4**	**5**	**6**	**7**	**8**	**9**	**10**	**11**	**12**	**13**	**14**	**15**
Use of pain medicine	1	1	200														
Breathing pain	2	4		65	*	14	5	4	7	3	7	6	*	3	4	6	3
Stress	3	4		*	40	24	*	4	3	3	4	4	3	6	3	4	7
Height	4	5		8	*	*	*	16	16	16	15	5	7	4	6		*
Physical activity	5	5		*	16	55	50	35	20	*	5	*	*	*	*		
Weight	6	13				*	15	18	18	17	13	8	4	4	6	6	6
Age [60,80)	7	11		*	5	7	10	*	16	13	17	7	*		6	*	*
Stomach pain	8	12		3	6	6	8	17	*	13	9	15	4	8	6	8	10
T2D	9	12			*	3	8	*	14	17	15	15	13	*	15	9	10
Breast pain	10	12		37	23	8	*	*	5	6	4	7	5	8	5	6	7
4th quartile income	11	17		*	9	16	13	5	3	6	4	9	4	10		3	5
Whiplash	12	17					*	6	7	7	10	14	14	6	9	10	7
Mild liver disease	13	15.5				*	*	5	9	5	8	10	15	14	9	7	14
Anxiety	14	14		18	25	9	6	3	3	9	*	7	7	4	6	*	5
BMI [30,35)	15	13			3	5	15	20	16	*	9	8	4	6	3	8	4
Arthritis	16	15				*	*	4	7	7	*	*	16	10	17	7	8
OA	17	15				*	*	4	8	8	10	*	13	16	13	*	17
Musclepain	18	17			5	5	7	5	4	5	9	9	10	*	8	8	4
Nervedamage	19	20.5					*				*	3	3	10	9	8	13
Higher education	20	20			*	*	7	*	3	7	10	*	7	7	5	7	3
Medium education	21	25									*	3		*		3	3
Non-mild livear disease	22	25					*	*	*	3	3	9	*	6	8	7	3
Smoker	23	22					*		4	*	*	*	5	6	4	13	*
Asthma	24	28				3	3	4	4		9	3	4	3	4	6	4
Non-smoker	25	26.5											*	*		3	*
Number of kids = 1	26	37.5				*		*	*	3	*	*	*	*	*	3	9
T1D	27	31										*	*	*	4	6	*
Lives alone	28	30				*		*	*	*	*		3	4	6	4	4
BMI [40, .)	29	39.5								*	*	*	4	*	4	5	5
Age [80, .)	30	40							*	3		*		*	*	*	5
COL	31	30								*			*	*	4	3	*
Backpain	32	33.5							*	*			3	3	4	4	*
Sore throat	33	32		*	*	*	*	3	4	*	3	*	*	6	4	3	*
Depression	34	35										*		*	*	*	
2nd quartile income	35	37										*		4	3	*	3
Female (CPR)	36	38					*	*	*	*							*
Other headache	37	37.5											*			*	
Hypertension	38	40											*	*	*	*	
Migraine	39	39						*	*	3	*	*	4		3	5	*
Age [40,60)	40	38			*	*	3	*	*	*			*	*	*	*	*
Income missing	41	39								*					*		3
Number of kids = 2	42	35											*		*	3	
Number of kids = 0	43	39					*	*	*	5	3	*	*	3	3	*	3
Shoulder/neck	44	42					*	*	*	*	*	3	*	*	*	*	3
Post operative syndrome	45	42													*	*	*
Asplenia	46	48															
Postoperative pain	47	45									*			*			
BMI [35,40)	48	38					*		3	3	4	4	3	5	3	3	7
Educational level missing	49	41							*		*		3	3	*		*
Malicious tumor	50	43													*	*	*
BMI [25,30)	51	42.5														*	*
Jointpain	52	45											*			*	
3rd quartile income	53	43											*		*	*	*
Other nervedisease	54	43.5															*
CKD	55	44															
Dementia	56	47															
Neurological symptoms	57	43														*	*
CVD	58	43.5															

count16	count17	count18	count19	count20	count21	count22	count23	count24	count25	count26	count27	count28	count29	count30	count31	count32	count33	count34	count35	count36
**16**	**17**	**18**	**19**	**20**	**21**	**22**	**23**	**24**	**25**	**26**	**27**	**28**	**29**	**30**	**31**	**32**	**33**	**34**	**35**	**36**
*	*	3	*	*	*	3	*	3	4	3			*		*	3	*		*	*
*	6	5	*	*	3	3	6			3	*	*	*		*					*
	*					*					3		*				*	*		
4	4	5	*	3		3	*	*	3	3	*	*	*	3	*		3	*		
4	*	*	*	*	*		*	*	*	*	*		*	*	*	*	*	4	*	
9	6	3	4	7	3	*	*	*	3	3	4	*	3		3	*	3	*	*	
5	*	5	5	3	4	3	4	*	*	3		*	*			*	*			
5	3	*	5	4	5	5	*	3	3	*	6	3	*	3	3	*	*	*	*	*
8	4	6	5	8	5	*	3		5	5		*	*	*	3	3		3	4	3
4	9	7	*	6	6	*	5	8	5	*	5		3	*	*	6	3		5	*
9	*	7	7	5	7	*	*	4	*	*	3	*	*	3	5			3	*	
3	8	*	3	5	7	*	8	4	3	*	4	5	5	*		3	*	*	*	3
4	3	8	*	*	*	*		*	*	3	*	*	*		*		4	*		*
*	7	7	10	7	5	7	3	7	3	*	5	*	*	5			*	*	3	*
17	10	5	10	4	4	*	9	*	*	*	*	*	*	4		*			*	*
8	7	3	8	7	*	*	4	3	6	6	*	3	*	*	5	*	*	3	4	4
4	13	*	*	*	6	5	8	5	6	10	4	3	8	3	5	3	4	5	*	*
6	4	7	6	10	10	8	8	5	6	*	4	8	4	7	3	5	*	3	4	4
7	5	*	10	7	8	6	*	*	*	7	4	3	15	9	7	*	6	4	5	4
4	*	*	6	4	5	7	9	3	6	8	9	4	4	*	5	7	*	4	*	5
*	10	6	8	7	8	10	6	*	9	8	4	*	14	6	*	5	*	*	*	*
6	*	4	*	4	8	4	5	4	3	7		6	6	*	4	6	4	6	3	9
3	9	9	7	9	8	10	9	*	10	8	*	15	10	8	9	4	9	4	4	4
3	*	6	*	3	6	4	3	*	5	3	3	5		4	3	*	5	5	*	3
*	*	5	5	5	6	8	4	5	*	*	6	8	5	6	6	5		5	4	7
*	*	6	8	5	9	8	*	4	7	6	3	6	3	8	5	3	9	*	8	5
5		4	*	5	4	*	*	6	4	3	3	5	*	*	*	*	4	*	5	6
*	*		7	*	3	*	5	*	8	3	8	*	4	5	*	3	7		3	*
5	5	6	6	7	9	5	13	5	3	3	10	3	4	7	7	8	6	8	7	6
5	4	*	5	8	*	4	3	7	*	6	4	6	3	4	3	*	4	7	*	3
*	*	5	*	4	4	*	4	4	6	3	*	3	5	*	4	3	6	6	5	4
3	*	3	3	5	*	5	5	4	5	6	9	7	4	5	5	8	5	6	6	5
*	*	*	3	*	*	*	5	*	6	5	5	*	5	10	3	7	4	5	4	3
	3	4	4	*	*	3	*	7	3		4	5	3	*	*	*	6	*	10	8
*	3	6	*	*	4	6	6	6	3	3	*	4	7	4	4	10	5	6	*	8
4		*	*	3	*	5	3	6	3	6	6	5	9	*	5	5	3	7	3	3
5	*		*	3	3	5	5	3	*	3	3	3		*	6	5	4	4	6	*
4	3	*	3	5	3	3	*	3	3	4	5	5	5		5	4	*	9		6
	3	3	*		3	7	5	3	5	4	6	6	*	6	4		6	5	*	5
*	*	*	6	*	3	3	*	*	6	3	6	5	4	7	9	8	6	5	7	6
*	*	*			*	4	*	*	5	6	*	4	4	4	4	4	7	3	3	6
*	3	4	*	*	3	*	3	*	*	3	3	*	3	4	*	*	6	7	5	*
	*			*	*	*	3	5	3	3	4	*	*	5	4	5	4	4	8	3
												*			*		*		4	4
3	*			*	*	*		*	*	*	4	3	*	*	4	3	3	7	5	6
4	*	*	*	*		*	*	*	4		4	*	4	3	*	5	*	5	5	*
*		3	3	4	3	3	*	5	*	3	*	*	6	4	*	6	*	4	4	*
*	*	*	*		*	*	3	*		4	*	5	3	*	3	7	*	3	7	5
*	4		*	4	3		*	*	*	3	*	5	3	*	8	4	8	*	3	10
		*	*		*	*		*	4	3	6	5	*	*	6	*	5	3	6	5
*	4	*	*	*	4		*	*	4	*	3	*	3	3	6	4	3	4	8	4
	*			3	*		3	5	3	*	4	3	3	9	3	5	5	4	5	*
*		*	*	*	3	*	*		*	*	*	5	5	6	5	5	7	8	*	5
					*	*	*	*		*	*	*	*	*	6	5	3	*	4	6
*	*	*		*	*	*	*	3	*	5	*	3	*	*	4	6	4	5	*	4
*	*						*	5	*	*	*	*	*	3	4	5	4	4	8	6

count37	count38	count39	count40	count41	count42	count43	count44	count45	count46	count47	count48	count49	count50	count51	count52	count53	count54	count55	count56	count57	count58
37	38	39	40	41	42	43	44	45	46	47	48	49	50	51	52	53	54	55	56	57	58
*	*							*		*	*				*		*				
		*				*	*											*			
		*													*						
*	*	4	*	3	*	*	*	*	3	*	3	5	4	*	*	*	*		*	*	
*	*		*	*	*		*	3	*		5	*	*	6	3	*	5	3	4	*	5
*	*		*	*	*	*	*						*		*		*			*	
*		*		*					*			*									
3	*	3	*	*			*										*				
3	3	*	3	3	*	*	*	3	*	*	3	*	*					*	*	*	3
*	*	*	*		*	*	*			*	*				*		*		*	*	
*	*					*			*		*	*		*	*			*		*	
*	*	4		*	3	*		*	*			*	*	*				*		*	
*	3	3	6	*	*	*	*	*	*	3	*	3	*	*	*	*	*	5		*	*
*				*	*		*		*	*				*							
*	*	*				*					*	*	*								
		4		*				3	*			5				*	*		*		
5	*	*	*	*		*		*		*		*	*		*			4		*	*
4	*	*		*		*	*	*			*										
7	3	*	*	*	*	*	*	*	*	4						*	*		*	*	*
*	*	5	4	3	*	4	3	*	*	*	5		*	*	*	*	3	*	*	*	*
4	*		*	3	*	*	*			*			*		4						*
3	4	*	*	4	5	7	3	*	*	3	3		3	*	*	*	*	*	3		3
*	3	4	4		*	*		*	*	*	*	*			*						
4	4	6	4	5	5	4	5	10	4	9	5	5	5	7	4	5	3	4	*	3	*
*	5	5	*	6	3	6	7	*	4	*	5	5	4	*	*	3	*	*	*	4	3
5	4	5	*	6	*	*	*	3	7	*	*	3	5	*	*	*	3	*	*	*	*
	6	3	9	4	8	6	5	6	5	6	4	5	6	7	8	*	*	5	7	3	3
4	5	6	6	6	3	*	6	7	5	4	10	7	5	5	4	7	3	8	6	6	3
*	*	*	*	5	4	*	*	*	4	5	3	*	*	4	*	4	4		5	*	*
4	7	3	8	5	9	4	5	4	3	5	*	4	*	6	3	*	*	*	4	5	*
5	8	3	3	3	*	3	3	6	3	3	*		6	*	5	3	3	5	*	5	4
*	3	7	3	3	3	4	5		5	3	7	4	8	*	5	*	7	4	7	6	*
8	4	4	3	8	4	4	6	5	3	7	5	4	4	8	3	6	6	5	3	5	*
*	10	7	4	*	4	5	5	3	3	4	*	7	3	*	8	*	7	9	6	4	3
4	4	9	8	7	3	7	7	5	4	4	8	3	*	4	3	*	3	4	4	4	5
*	3	6	4	7	8	9	7	7	6	*	3	3	5	10	3	8	4	4	4	*	5
3	5	4	7	6	9	*	5	4	7	5	5	3	3	7	6	*	7	6	6	6	3
4	*	3	7	3	4	8	4	5	3	8	5	5	4	6	7	4	4	5	6	4	4
4	8	8	*	6	6	*	4	4	9	5	5	3	5	8	5	5	8	4	7	6	5
7	*	5	4	*	3	*	7	9	7	8	8	*	5	3	3	4	6	*	*	*	*
3	3	6	3	5	3	10	9	7	3	*	*	*	4	5	5	7	6	4	7	*	*
6	4	*	7	5	8	7	*	6	8	6	10	6	6	5	*	5	7	6	5	5	9
10	8	3	3	4	9	5	9	5	7	8	3	7	8	3	5	10	*	6	4	10	5
5	5	*	4	8	5	*	8	8	8	8	5	9	6	7	16	6	6	3	5	7	5
4	4	4	7	*	7	6	9	7	7	7	6	3	6	5	16	6	9	9	8	6	9
*	*	5	7	6	*	5	3	5	*	5	3	7	8	*	5	3	4	4	5	6	*
4	5	6	8	6	6	4	4	8	4	4	5	7	6	7	7	7	5	*	6	*	7
7	5	4	3	4	3	7	4	9	8	4	9	6	5	5	8	6	3	10	6	14	*
8	3	7	4	6	3	9	3	6	9	10	10	4	8	8	*	5	9	6	3	6	3
*	*	5	3	5	8	7	10	3	9	6	5	*	9	4	7	8	4	7	9	10	8
8	3	4	4	6	8	6	*	4	5	6	5	6	7	3	4	9	15	3	8	9	9
6	8	3	10	4	6	3	8	3	9	7	7	6	3	9	5	10	*	8	5	7	*
4	4	5	6	3	9	*	7	5	4	5	5	3	4	9	10	6	5	7	*	13	7
5	3	7	9	6	6	7	5	4	7	6	6	14	10	5	8	*	9	9	13	5	5
5	7	9	5	5	6	13	9	6	5	7	4	3	9	6	5	7	7	10	5	7	7
4	9	4	9	*	7	7	*	9	4	8	6	8	8	8	3	13	3	8	8	8	4

Non-pre-COVID pain group

dendrogram_text	original_order	median	count1	count2	count3	count4	count5	count6	count7	count8	count9
**Predictor**	**Original order**	**Median**	**1**	**2**	**3**	**4**	**5**	**6**	**7**	**8**	**9**
**Stress**	1	1	194	3	3						
**Female (CPR)**	2	2	*	104	10	14	44		6	16	4
**Weight**	3	3		*	104	38	17	39			
**Higher education**	4	4		8		136	*	18	*	10	3
**Age [40,60)**	5	5		*	83		*	15	*		
**4th quartile income**	6	6	4	*		7	5	33	17	28	17
**Physical activity**	7	7				*	27	67	93	*	
**Height**	8	8				3	26	9	47	*	37
**Asthma**	9	9							*	50	85
**Neurological symptoms**	10	10								23	43
**Anxiety**	11	13									6
**Medium education**	12	13									4
**Age [60,80)**	13	13					27	19	*		
**BMI [40, .)**	14	14									
**Non-smoker**	15	17									
**BMI [25,30)**	16	17									
**Lives alone**	17	18									*
**Non-mild livear disease**	18	19									
**CVD**	19	19.5									
**BMI [30,35)**	20	22				*					
**Depression**	21	21									
**T2D**	22	21									
**Smoker**	23	22									
**Number of kids = 0**	24	24									
**3rd quartile income**	25	24									
**Age [80, .)**	26	25									
**Number of kids = 2**	27	30									
**Number of kids = 1**	28	30									
**Mild liver disease**	29	28									
**Asplenia**	30	31									
**COL**	31	33									
**BMI [35,40)**	32	29.5									
**T1D**	33	34.5									
**Dementia**	34	33									
**Malicious tumor**	35	35									
**Educational level missing**	36	34									
**CKD**	37	36									
**2nd quartile income**	38	36									
**Hypertension**	39	34									
**Post operative syndrome**	40	34									
**Income missing**	41	35									

count10	count11	count12	count13	count14	count15	count16	count17	count18	count19	count20	count21	count22	count23	count24	count25
**10**	**11**	**12**	**13**	**14**	**15**	**16**	**17**	**18**	**19**	**20**	**21**	**22**	**23**	**24**	**25**
*															
*	8	18	6	9	3					*					
7															
15	*														
40	8	3	*												
86	*	*	*	*	*						*				
16	49	24	24	16	9	*	5	5	6	*	7	*	5	*	*
18	34	*	34	*	15	13	6	5	*	*	4				*
7	*	17	35	17	13	6	5	4	5		*	*	7	*	
	10	27	24	*	16	10	7	5		*		4	*		*
*	8	16	14	15	24	19	*	19	*	*	6	8	*	9	*
*	4	10	10	*	25	*	*	14	*	*	*	7	*	4	7
3	*	14	10	*	19	*	16	*	*	*	15	17	6	4	3
	*	7	10	3	18	17	24	13	15	14	17	6	9	8	6
	*	*	4	*	10	10	15	*	27	19	9	13	*	6	14
	*		3	7	8	14	14	16	6	10	14	16	14	19	7
*	7	18	7	3	10	9	7	10	*	*	*	16	9	4	*
*			7	3	5	17	9	14	15	14	17	15	14	13	*
				3	5	8	19	*	18	16	15	17	13	9	7
		*	*	*	4	10	10	8	9	13	15	9	*	9	*
*	3	*	4	*	6	4	9	15	*	16	6	*	7	19	10
				*		5	7	6	7	15	15	13	14	*	*
								*		*	4	6	5	8	5
						*	*	*	3	4	5	5	3	7	9
			*	*	*	*	3	5	8	4	*	5	19	*	13
								*		*	3	*	3	10	14
		*						4		4	*	*	8	4	6
					5	6	6	4	*	6	6	5	3	*	10
								*			*	4	*	3	3
					*	*	*		6	*	4	*	7	5	6
						*					*	3	*	*	5
								*	*	4	*	4	5	*	8
								*		*		*	*	5	6
							*		*	*	*		*	4	*
								*				3	*	4	3
							*	*	*	*	3	*	3	4	*
									*				*		4

count26	count27	count28	count29	count30	count31	count32	count33	count34	count35	count36	count37	count38	count39	count40	count41
**26**	**27**	**28**	**29**	**30**	**31**	**32**	**33**	**34**	**35**	**36**	**37**	**38**	**39**	**40**	**41**
*		*				3	*			*		*			
	*														
3	*	3	*	*				*							
*		5	3	5	4	*		4	3	4	6	5	3	5	*
*	4	5		*	*	*									
*	4	3	*	3	*		*		*						
*	8	3	*		*	*	*	*							
4	8	3	4	3	*	*		*		*			*		*
6	8	5		*	*	3			*		*				
9	*	5	5	3	3	*	3			4		*	3		*
*	3	3	5	8	*	6	4	*	*	*	*		*	3	*
9	9	4	8	*	*	4		4	*	*		*			
15	5	3	*	4	9	*	*	*		*		*		*	
8	13	14	8	*	6	8	4	3		6	3	*	*		
10	9	7	*	4	3	6	8	4	5	*	*	*	3	5	3
13	13	*	10	9	7	6	*	3	3	3	3	3	4	*	*
14	15	20	18	14	20	17	9	14	13	5	*	*	4	*	*
8	*	*	18	18	9	*	20	6	*	10	8	3	5	4	4
5	15	8	13	8	10	6	7	10	7	9	7	6	*	7	4
8	7	8	6	*	8	*	*	6	*	5	8	8	16	6	4
8	4	9	9	9	8	*	15	*	17	8	18	10	*	13	7
7	7	7	5	10	10	7	6	13	5	5	*	6	9	13	5
8	6	7	7	8	9	*	10	18	*	9	13	*	19	14	*
*	4	8	8	8	14	8	7	5	9	15	7	15	14	18	15
*	7	9	*	7	9	6	13	13	15	14	18	18	*	14	19
9	4	6	10	9	9	8	7	13	*	13	*	*	13	*	14
3	4	5	6	3	6	15	9	14	17	17	18	*	23	15	8
7	3	*	3	*	*	6	*	10	17	28	16	23	14	*	4
3	*	13	8	9	*	8	*	14	9	10	16	16	20	*	14
6	6	7	8	*	*	15	9	8	17	17	14	*	7	19	*
4	7	4	*	4	13	*	15	19	*	10	16	*	14	14	19
